# DRP1-mediated mitochondrial shape controls calcium homeostasis and muscle mass

**DOI:** 10.1038/s41467-019-10226-9

**Published:** 2019-06-12

**Authors:** Giulia Favaro, Vanina Romanello, Tatiana Varanita, Maria Andrea Desbats, Valeria Morbidoni, Caterina Tezze, Mattia Albiero, Marta Canato, Gaia Gherardi, Diego De Stefani, Cristina Mammucari, Bert Blaauw, Simona Boncompagni, Feliciano Protasi, Carlo Reggiani, Luca Scorrano, Leonardo Salviati, Marco Sandri

**Affiliations:** 1 Venetian Institute of Molecular Medicine, via Orus 2, 35129 Padova, Italy; 2 Department of Biomedical Science, University of Padova, via G. Colombo 3, 35100 Padova, Italy; 3 Myology Center, University of Padova, via G. Colombo 3, 35100 Padova, Italy; 4 Department of Biology, University of Padova, via U. Bassi 58B, 35121 Padova, Italy; 5 Clinical Genetics Unit, Department of Woman and Child Health, University of Padova, via Giustiniani 3, 35128 Padova, Italy; 6 IRP Città della Speranza Corso Stati Uniti 4, 35127 Padova, Italy; 7 Department of Medicine - DIMED, University of Padova, 35128 Padova, Italy; 8 Center for Research on Ageing and Translational Medicine (CeSI-MeT)), via Luigi Polacchi, University G. d’ Annunzio, 66100 Chieti, Italy; 9 Institute for Kinesiology Research, Science and Research Center of Koper, Koper, Slovenia; 10 Department of Medicine, McGill University, Montreal, Canada

**Keywords:** Mitophagy, Energy metabolism, Skeletal muscle

## Abstract

Mitochondrial quality control is essential in highly structured cells such as neurons and muscles. In skeletal muscle the mitochondrial fission proteins are reduced in different physiopathological conditions including ageing sarcopenia, cancer cachexia and chemotherapy-induced muscle wasting. However, whether mitochondrial fission is essential for muscle homeostasis is still unclear. Here we show that muscle-specific loss of the pro-fission dynamin related protein (DRP) 1 induces muscle wasting and weakness. Constitutive *Drp1* ablation in muscles reduces growth and causes animal death while inducible deletion results in atrophy and degeneration. *Drp1* deficient mitochondria are morphologically bigger and functionally abnormal. The dysfunctional mitochondria signals to the nucleus to induce the ubiquitin-proteasome system and an Unfolded Protein Response while the change of mitochondrial volume results in an increase of mitochondrial Ca^2+^ uptake and myofiber death. Our findings reveal that morphology of mitochondrial network is critical for several biological processes that control nuclear programs and Ca^2+^ handling.

## Abstract (web summary)

Muscle loss is associated with altered expression of proteins involved in mitochondrial homeostasis, but whether this is causative remains unclear. Here, the authors show that genetic ablation of the pro-fission protein DRP1 leads to accumulation of abnormal mitochondria that induce muscle atrophy by altering Ca^2+^ homeostasis and cellular stress responses.

## Introduction

The maintenance of a functional mitochondrial network is particularly important for tissues that are highly structured and metabolically active, such as neurons, cardiac, and skeletal muscles. These tissues are constituted by post-mitotic cells that do not divide. As opposed to the tissues with high cell turnover that can dilute the damaged mitochondria among the dividing cells, the post-mitotic tissues use the fusion/fission machinery to preserve or restore mitochondrial function. Eventually, mitochondria that are irreversibly damaged are removed by the autophagy system^1^. Mitochondrial morphology and the number depend on the balance between two opposing processes, fusion and fission. Fusion induces the formation of an extended interconnected mitochondrial network enabling the mitochondria to mix and re-distribute their contents of metabolites, proteins, and mitochondrial DNA (mtDNA)^2^. An interconnected mitochondrial network prevents the local accumulation of defective/abnormal mitochondria which is advantageous under conditions of high energy demand, such as exercise^3,4^. Conversely, mitochondrial fission or fragmentation is a mechanism that segregates components of the mitochondrial network, which are dysfunctional or damaged, allowing their removal via mitophagy^5,6^. Hence, dynamic regulation of the fission–fusion events adapts mitochondrial morphology to the bioenergetic requirements of the cell. A failure of these systems predisposes to organ dysfunction and degeneration.

Mitochondrial fission depends on the cytosolic GTPase dynamin-related protein 1 (DRP1), which is recruited to the outer mitochondrial membrane and oligomerizes to form active GTP-dependent mitochondrial fission sites^7^. The fission machinery is critical for tissue development and function, and indeed constitutive *Drp1* knockout animals are embryonically lethal^8^. Consistent with this crucial role of mitochondrial dynamics in post-mitotic organs, conditional deletion of *Drp1* in the brain leads to perinatal lethality due to brain hypoplasia^8^. The severe phenotypes of these mice preclude studies of mitochondrial function in adult organs^8,9^.

Striated muscles are post-mitotic tissues that rapidly respond to metabolic challenges, like exercise, feeding, or fasting. The plasticity of the mitochondria is a key factor in myofiber metabolic adaptation to different conditions^6^. Alterations in the content, shape, or function of the mitochondria are associated with muscle wasting. Overexpression of the mitochondrial fission machinery in adult muscle is sufficient to induce muscle atrophy^10,11^. In elderly individuals, mitochondria are abnormally enlarged, more rounded in shape, and display matrix vacuolization and shorter cristae^12^. We have recently found that fusion and fission proteins are greatly reduced in atrophic muscles of old sedentary people while they are normally expressed in hypertrophic muscles of senior sportsmen^13^. Similarly, DRP1 protein is reduced in cardiac and skeletal muscles during ageing in mice^14^. Consistently, a transcriptomic and proteomic study in rats has revealed a correlation between reduced expression of fusion/fission proteins and age-related muscle loss^15^. The decrease of fusion and fission proteins happens also in several inherited and acquired muscle diseases. In cancer cachexia and in chemotherapy-induced cachexia, DRP1, OPA1, and Mitofusin2 are dramatically suppressed^16–18^. The same pattern of reduction has been found in an animal model of Myasthenia gravis^19^. Finally, loss-of-function mutation in the DRP1 receptor, MiD49, causes severe myopathy in humans^20^. Altogether these observations suggest that changes of mitochondrial shape might be an important player in determining muscle mass maintenance, homeostasis, and metabolism. However, little is known about the requirement of mitochondrial fission and the downstream signaling in skeletal muscle in vivo. To understand the physiological relevance of the fission machinery in muscle homeostasis, we generate two mouse models lacking *Drp1* in skeletal muscle and show that these mice develop a lethal mitochondrial myopathy caused by activation of protein breakdown, ER stress, hypoglycemia, and abnormalities in Ca^2+^ homeostasis.

## Results

### Muscle-specific *Drp1* deletion results in postnatal lethality

**Fig. 1:**
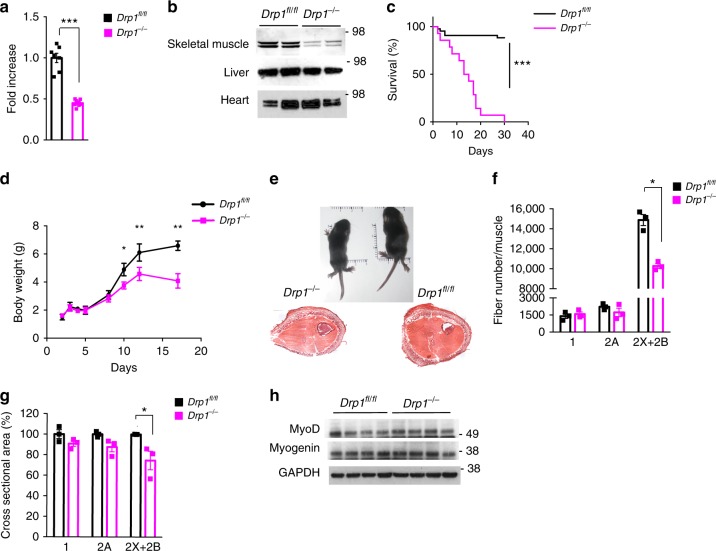
DRP1 deletion impairs muscle growth and causes a lethal phenotype. *Drp1* mRNA (**a**) and DRP1 protein levels (**b**) are downregulated only in skeletal muscle and not in other tissues in *DRP1*-null mice. Each condition represents the average of at least three independent experiments ± SEM. **c** Kaplan–Meier survival curve of *Drp1*^*fl*/*fl*^ and *Drp1*^−/−^ littermates (WT, *n* = 43; KO, *n* = 14), indicates that muscle-specific *DRP1* deletion results in lethality within postnatal day 30. **d** *DRP1*-deficient mice show an impairment of total body weight. **e** Upper panel: representative photograph of control and KO littermates at postnatal day 12 showing that *Drp1*^−/−^ mice are smaller than controls. Lower panel: haematoxilin–eosin (HE) staining of hindlimb cross-section. The cross-sectional area analysis of fibers (**g**) and the total number of different fiber types in hindlimb muscles (**f**) indicate a reduction of *DRP1* KO fiber size without hypoplasia. The data represent mean ± SEM (**g**, *n* = 3 and **f**, *n* = 3). **h** Representative immunoblot analysis of muscle homogenates of four independent experiments. MyoD and Myogenin were normalized to GAPDH expression levels. The data represent average ± SEM. Two-tailed unpaired Student’s *t* test was used. Statistical significance: *p ≤ 0.05; **p ≤ 0.01; ***p ≤ 0.001

Because DRP1 level decreases in muscle during ageing and in different conditions of muscle wasting^13,14,16–18,20^, we generated a muscle-specific *Drp1* knockout mouse by crossing the *Drp1*^*fl*/*fl*^ mice with MLC1f-Cre transgenic mice (*Drp1*^−/−^), where Cre recombinase is expressed in differentiated muscle cells and not in muscle stem cells or in myocytes^21^. Muscles of *Drp1*^−/−^ newborns showed a decrease of DRP1 transcript and protein level (Fig. 1a, b; Supplementary Fig. 1a–c), while it was normally expressed in other tissues, including the heart (Fig. 1b). Immunofluorescence analyses confirmed the absence of DRP1 protein in myofibers irrespectively of the type of muscle (Supplementary Fig. 1a, b), while interstitial cells, skin, and vessels do express DRP1 and contribute to the protein detected in western blot of Fig. 1b. Importantly, these mice died within 30 days of postnatal life (Fig. 1c). During the first 17 days of postnatal life, the body growth was reduced in *Drp1*^−/−^ mice (Fig. 1d, e). Muscles displayed a reduction in the total number of fibers (Supplementary Fig. 1e) because glycolytic type 2B/2X fibers are less than controls (Fig. 1f) and a concomitant decrease of the cross-sectional area (Fig. 1g). The reduced number of fibers prompted us to analyze myogenesis. However, MyoD and myogenin expression, two markers of muscle stem cell proliferation and differentiation, did not differ between *Drp1*^*−*/−^ and control mice (Fig. 1h; Supplementary Fig. 1d), suggesting that myogenesis is not perturbed at neonatal stage despite postnatal myofiber growth is affected by *Drp1* deletion.

### *Drp1* deletion impairs mitochondrial function

**Fig. 2:**
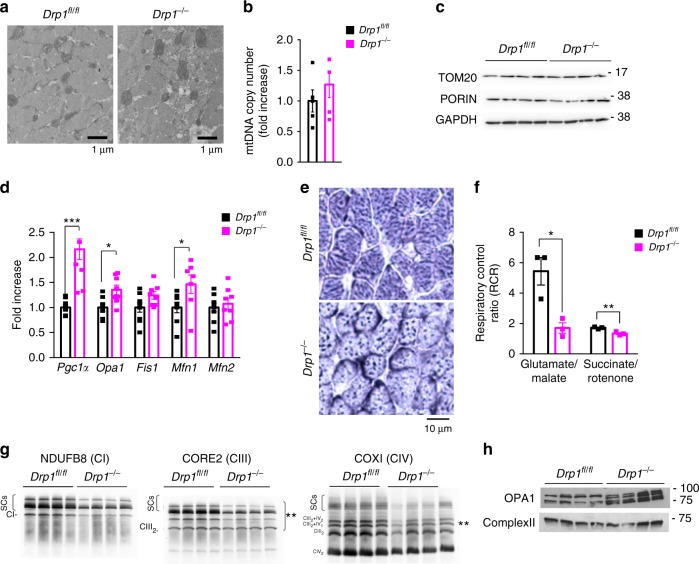
Muscle DRP1-chronic inhibition alters mitochondrial morphology, RCS organization and complex-I- and complex-II-dependent respiration. **a** Representative EM cross-sectional images of mitochondrial distribution and morphology in gastrocnemius from WT and *Drp1*^−*/*−^ mice. In cross-sections, the different morphology of mitochondria in *Drp1*^*−/*−^ from WT is often evident: in *Drp1*^−*/−*^ fibers mitochondria are more often larger in size (scale bar: 1 μm). **b** Mitochondrial DNA copy number quantification. mtDNA was amplified by RT-PCR from the total DNA of gastrocnemius muscles of the indicated genotype. The data are normalized to control and represent average ± SEM of five independent experiments. **c** Representative immunoblots of four independent experiments of muscle homogenates showing no difference of porin/GAPDH ratio. **d** RT-PCR analysis of mitochondria dynamics transcripts of muscles of WT and Drp1^−/−^. DRP1 deletion induces PGC1α, OPA1, and Mfn1 transcript levels. The data represent average ± SEM (*n* = 8) and are normalized for GAPDH and expressed as fold increase of controls. **e** Succinate dehydrogenase (SDH) staining of *Drp1*^*fl*/fl^ and Drp1^−/−^ muscles show a different distribution of mitochondrial network in KO mice. **f** Respiratory control ratio (RCR) of muscle-isolated mitochondria energized with 5 mM/2.5 mM GLU/MAL or 10 mM SUCC. The data represent average ± SEM of three independent experiments. **g** DRP1 deficiency leads to a significant reduction of CIII and CIV assembly in respiratory chain supercomplexes (RCS). Representative Blue Native PAGE analysis showing RCS developed and normalized for individual respiratory chain complexes. Left panel: CI subunit NDUFB8, middle panel: CIII-Core2 protein 2 and right panel: CIV subunit COXI. **h** Representative immunoblot analysis of OPA1 different isoforms show an increase in OPA1 cleavage in Drp1-null muscles (*n* = 4). Two-tailed unpaired Student’s *t* test was used. Statistical significance: *p ≤ 0.05; **p ≤ 0.01; ***p ≤ 0.001

To understand whether DRP1 inhibition altered mitochondria network, we monitored the mitochondrial ultrastructure by electron microscopy (EM) (Fig. 2a). The quantitative EM analysis revealed that, while the relative volume occupied by the mitochondria was not significantly different in DRP1-deficient muscle (Supplementary Fig 2a), the average size of individual organelles was larger in *Drp1*^−/−^ muscle fibers than controls. These findings indicate that DRP1 ablation results in remodeling of the mitochondrial network with bigger, but fewer, mitochondria with no changes in the relative fiber volume occupied by the mitochondrial network (Supplementary Fig. 2a). Consistently, the mitochondrial DNA content was not changed between the two genotypes (Fig. 2b), suggesting that the total mitochondrial content was not decreased. Similarly, mitochondrial mass revealed by TOM20 and porin proteins was not affected by DRP1 deletion (Fig. 2c; Supplementary Fig. 2e). Lack of DRP1 induced an upregulation of the profusion genes *Pgc1*α, *Mfn1*, and *Opa1* (Fig. 2d) that may contribute to the observed increase of the mitochondrial volume. SDH staining of neonatal muscles confirmed the presence of smaller fibers containing an abnormal mitochondrial network with bigger and fewer puncta in *Drp1*^*−/−*^ (Fig. 2e). Functionally, respiration was reduced in the mitochondria purified from *Drp1*^*−/−*^ muscles (Fig. 2f; Supplementary Fig. 2b) being complex-I-driven respiration the most severely affected. Consistent with the respiration data, the measurements of complexes activity showed a significant reduction of complex-I and III (Supplementary Fig. 2c). Immunoblots of WT and *Drp1*^−*/*−^ mitochondrial proteins revealed a downregulation in the levels of the tested respiratory chain complexes subunits (Supplementary Fig. 2d). Finally, supercomplexes, separated by Blue Native PAGE, were decreased (Fig. 2g; Supplementary Fig. 2f). Since supercomplex formation is regulated by OPA1 levels^22^, we tested OPA1 and showed that its processing was increased in the *Drp1*^*−/−*^ mitochondria, where short isoforms accumulated (Fig. 2h) in a picture similar to cells where DRP1 is chronically depleted^23^. Therefore, DRP1 ablation alters mitochondrial morphology and function.

### *Drp1* deletion reduces protein synthesis and induces atrophy

**Fig. 3:**
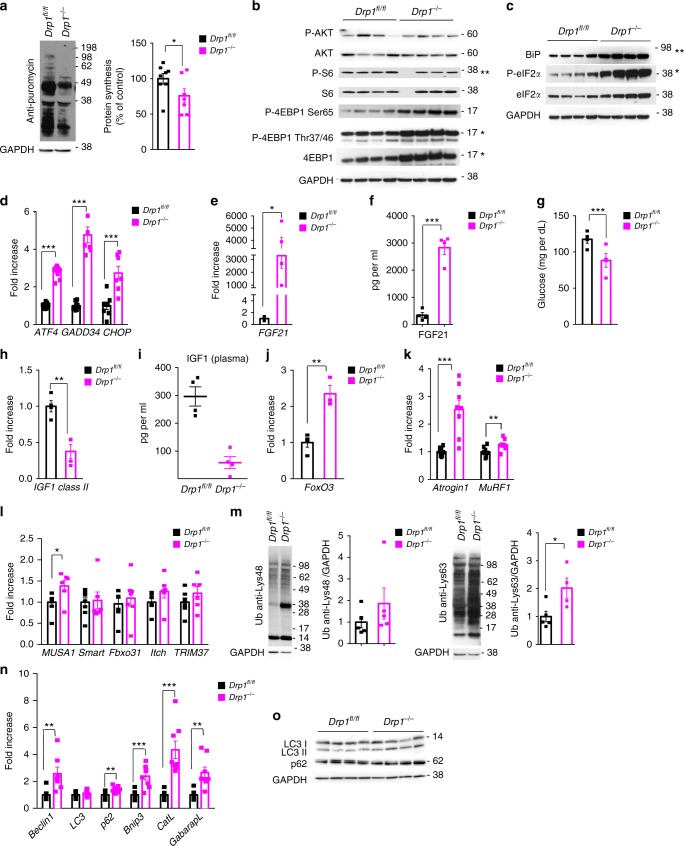
DRP1 absence leads to downregulation of protein synthesis and activation of the Ubiquitin–proteasome and autophagy–lysosome pathways. **a** In vivo SUnSET technique showed a significant reduction of protein synthesis in DRP1-ablated mice muscles. Representative western blot and quantification of the puromycin-labeled peptides, expressed as percentage of the values obtained in the control group, are depicted. The data represent average ± SEM of eight independent experiments. **b** The total protein extracts from newborns muscles were immunoblotted with the indicated antibodies. A representative immunoblot of four independent experiments is shown. Statistical significance of specified densitometric ratios is indicated on the right. The data are normalized to GAPDH (*n* = 8). Representative immunoblot (**c**) and RT-PCR (**d**) showing the activation of the unfolding protein response (UPR) pathway in DRP1 KO muscles. The data represent average ± SEM of seven independent experiments. **e** RT-PCR analysis of transcriptional levels of muscle FGF21 (*n* = 3). **f** Quantification of blood levels of FGF21 shows an increase in DRP1 KO mice (*n* = 3), leading to a decrease in blood glucose (**g**, *n* = 4) and to a significant decrease in IGF1 class II mRNA level in the liver (**h**, *n* = 4). The data represent average ± SEM. **i** Quantification of IGF1 in plasma from *Drp1*^*−/*−^ and control mice shows a significant decrease of IGF1 circulating levels in the absence of DRP1. RT-PCR analysis of FoxO-dependent transcripts shows an increase in FoxO3 levels (**j**), in the muscle-specific ubiquitin ligases Atrogin1 and MuRF1 (**k**) and in the novel Ubiquitin–ligase MUSA1 (**l**). The data represent average ± SEM (*n* = 6 per condition). **m** Representative blots and densitometric analysis of at least four independent experiments of total muscle extracts immunoblotted for anti-Ubiquitin (Lys48) and for anti-Ubiquitin (Lys63) normalized to GAPDH. The data represent average ± SEM. **n** Quantitative PCR analysis of autophagy-related transcripts showing a significant induction of autophagy markers in Drp1^−/−^. The data represent average ± SEM (*n* = 8 per condition). **o** Representative immunoblots of four independent experiments of autophagy-related proteins. The data represent average ± SEM (WT, *n* = 4; KO, *n* = 6). Two-tailed unpaired Student’s *t* test was used. Statistical significance: *p ≤ 0.05; **p ≤ 0.01; ***p ≤ 0.001

The observed mitochondrial dysfunction does not mechanistically explain the impaired animal growth of *Drp1*^*−/−*^ mice. During the first 2 weeks of postnatal life, muscle growth depends on fusion of muscle stem cell to the growing myotube and on protein synthesis^24^. Since muscle stem cell differentiation was not perturbed during neonatal growth (Fig. 1h; Supplementary 1d), we monitored protein synthesis in vivo by using the SUnSET technique^25^. Importantly, protein synthesis was reduced by 30% in *Drp1*^*−/−*^ mice (Fig. 3a; Supplementary Fig. 3a). We therefore turned to pathways that regulate translation. Insulin signaling, through mTOR, plays a critical role in controlling protein synthesis. While AKT phosphorylation was not affected, two mTOR downstream targets were changed, but in opposite pattern. One mTOR-dependent phosphorylation site of 4EBP1 (Thr 37/46) was increased, while a second one (Ser 65) showed a trend of increase. Conversely, a second downstream mTOR target, S6, was less phosphorylated in *Drp1*^−*/*−^ (Fig. 3b; Supplementary Fig. 3b). Thus, the AKT/mTOR/4EBP1/S6 axis is not consistent and does not explain the reduction of protein synthesis, suggesting that other regulators are involved in the translation impairment. The unfolded protein response pathway through the kinase PERK phosphorylates eIF2α, a critical ribosome assembly initiation factor. When eIF2α is phosphorylated, the ribosomal 43S complex formation is decreased, ribosome assembly is impaired, and general protein synthesis is reduced. Interestingly, levels of p-eIF2α and of the chaperone Bip/Grp78, a downstream target of UPR, were increased in *Drp1*^−*/−*^ muscles (Fig. 3c; Supplementary Fig. 3b). Moreover, PERK-dependent UPR triggers the activation of the transcription factor *ATF4* that transcribes several genes, including *BiP*, *GADD34*, and *CHOP*. All these genes, including *ATF4*, were induced in knockout mice, supporting the concept that UPR is strongly activated in *Drp1*^−*/*−^ muscles and can account for the translation impairment (Fig. 3d). Consistently, *FGF21*, a fasting-mimetic cytokine under *ATF4* regulation, was induced (Fig. 3e), resulting in increased FGF21 blood levels and decreased glycemia in fed *Drp1*^−/−^ mice (Fig. 3f, g). Because FGF21 causes GH resistance, we tested liver expression of IGF1, the critical anabolic hormone that controls the tissues size during postnatal growth. *IGF1* transcript level was downregulated in hepatocytes (Fig. 3h), and consistently, the IGF1 blood level was also reduced in knockout mice (Fig. 3i). The decrease of IGF1 explains the reduced size of DRP1-deficient mice.

Since the size of the cell is determined by the balance between protein synthesis and degradation, we also monitored the atrophy-related pathways^26,27^. Protein breakdown is controlled by a transcriptional-dependent program that regulates the expression of a subset of genes named atrophy-related genes. These genes encode for enzymes that catalyze rate-limiting steps during ubiquitination process and autophagosome formation. FoxO3 is the master regulator of the atrophy program, and its activity is controlled at multiple levels, including its transcription. Interestingly, FoxO3 was induced in *Drp1*^−/−^ muscles (Fig. 3j) and its downstream targets, the atrophy-related ubiquitin ligase *atrogin-1* and *MuRF1*, were upregulated (Fig. 3k). We also measured a new set of ubiquitin ligases that we have identified to be under FoxO regulation and involved in muscle atrophy^26,28^. Among them, *MUSA1* was upregulated in *Drp1*^−/−^ mice (Fig. 3l). According to the induction of the ubiquitin ligases, also the amount of lysine 48 and lysine 63 polyubiquitinated proteins was increased in *Drp1*^−/−^ mice (Fig. 3m). Since the autophagy–lysosome system is also induced during muscle atrophy and is controlled by FoxO3^29^, we measured expression of FoxO-dependent autophagy-related genes^26^. Several genes were induced in knockout mice (Fig. 3n) confirming that an atrophy program was activated in these mice. However, the ratio of LC3II on GAPDH was only mildly increased, while p62 was not affected (Fig. 3o; Supplementary Fig. 3b), suggesting that autophagy was weakly induced at 12 days of postnatal life. In conclusion, DRP1 deletion led to a defect of muscle growth caused by a fasting-like condition, inhibition of protein synthesis, and mild induction of the ubiquitin–proteasome and autophagy–lysosome-dependent protein breakdown.

### Acute deletion of *Drp1* causes muscle wasting and weakness

**Fig. 4:**
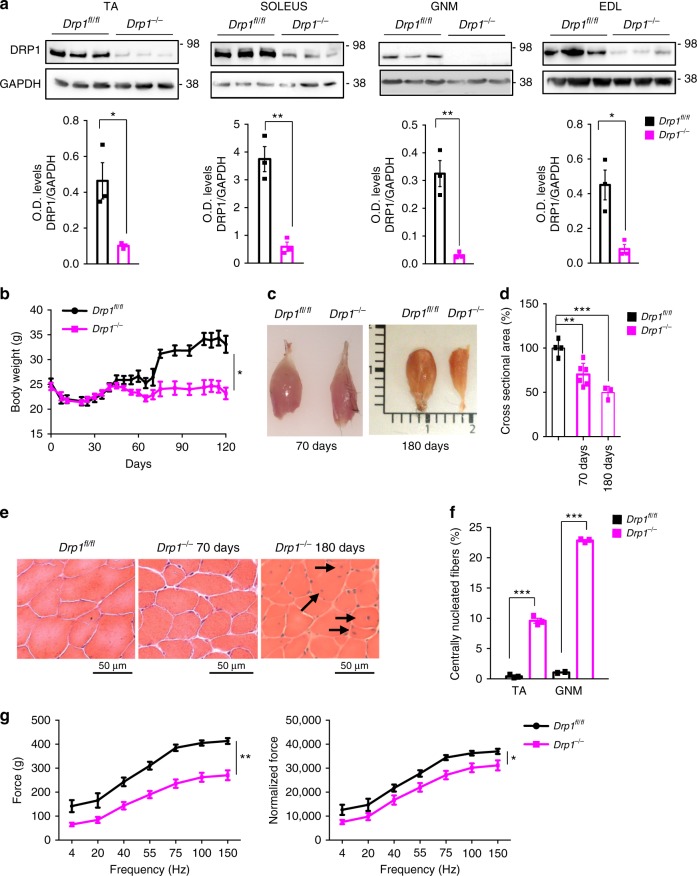
DRP1 loss in adult animals causes body weight loss, muscle atrophy and muscle weakness. **a** DRP1 protein levels are downregulated in different types of skeletal muscles in HSADRP1-null mice. O.D. levels represent the average of at least three independent experiments ± SEM. **b** Growth curve of control and *Drp1*^−/−^ littermates during and after tamoxifen treatment. KO mice start to lose body weight after 7 weeks of tamoxifen treatment and during the following weeks. The data represent average ± SEM (WT, *n* = 10; KO, *n* = 11). **c** Dissected gastrocnemius muscles from the control and DRP1-null mice show an important muscle atrophy after DRP1 deletion in adult animals. **d** Quantification of the cross-sectional area of myofibers indicates a significant reduction in DRP1-ablated muscles. Values represent average ± SEM (WT, *n* = 5; KO 70 days, *n* = 5; KO 180 days, *n* = 3). **e** Representative haematoxilin–eosin staining of Tibialis Anterior muscle showing signs of myofiber degeneration/regeneration in *Drp1*^−/−^ after 180 days of treatment. **f** In Drp1^−/−^ muscles after 180 days of treatment, there are 10 and 25% of myofibers are centrally nucleated, in tibialis anterior muscle and gastrocnemius muscle, respectively (*n* = 3 mice each condition). **g** Force measurements performed in vivo on gastrocnemius muscles. Absence of DRP1 leads to a significant decrease in both absolute force and maximal specific force generated during tetanic contraction. The data represent average ± SEM (*n* = 3). Two-tailed unpaired Student’s *t* test and two-way analysis of variant (ANOVA) were used. Statistical significance: *p ≤ 0.05; **p ≤ 0.01; ***p ≤ 0.001

The severe phenotype of *Drp1*^−/−^ mice hindered further analyses in adulthood. We therefore generated a tamoxifen-inducible muscle-specific *Drp1* knockout mouse (*Drp1*^−/−^) by crossing *Drp1*^fl/fl^ mice with HSA-Cre-ER transgenic mice. Tamoxifen treatment in 5-month-old mice efficiently reduced DRP1 protein in different muscles (Fig. 4a). Within 50 days from tamoxifen treatment, mice started to differ in body weight when compared with controls (Fig. 4b). The reduction of body weight was secondary to important decrease of muscle mass. Skeletal muscles were smaller (Fig. 4c), and fiber size of *Drp1*^−/−^ was reduced by 25 and 50% when compared with controls at 70 and 180 days after the treatment (Fig. 4d). Concomitantly, there is also a decrease of white adipose tissue (Supplementary Fig. 4a). While fiber-type distribution was not affected at 40 days, an increase of 2X fibers and a concomitant trend of decrease of type 2A and 2B fibers were detected at 70 days (Supplementary Fig. 4b). Morphological analyses showed pure atrophy with no features of myofiber degeneration at 70 days (Fig. 4e). The decrease of fiber size was detected in all the different fiber types (Supplementary Fig. 4c, d). Moreover, muscle loss in DRP1-deficient mice was enhanced when nutrients were removed for 24 h, a time that does not induce significant myofiber atrophy in control mice (Supplementary Fig. 4e). Therefore, inhibition of DRP1 sensitizes muscle cells to the atrophy program. However, prolonged DRP1 deletion caused myofiber death as revealed by the appearance of center-nucleated fibers (Fig. 4e, f). This myopathic phenotype is evident at 180 days, but it appears at 120 days from treatment. Quantification of center-nucleated fibers confirmed that degeneration followed by regeneration happened in 10 and 25% of myofibers in TA and gastrocnemius muscles, respectively (Fig. 4f). To understand whether muscle performance was affected, we measured the strength of gastrocnemius muscle in living mice at 70 days after tamoxifen treatment when muscles display pure atrophy and do not show center-nucleated fibers. Single twitch and maximal absolute force were reduced (Fig. 4g), suggesting a loss of contractile proteins. However, the specific force, the force normalized for muscle mass, was also decreased in knockout mice (Fig. 4g). This finding suggests that, besides a decrease of myosin–actin content, there is also an impairment in force generation. We also measured muscle force and fatigue, ex vivo, in two other different muscles, EDL and Soleus. Interestingly, while muscle force was decreased during twitch and tetanic contractions in EDL, at 70 days, and in Soleus, already at 40 days after treatment, the fatigue was improved in EDL and Soleus of DRP1-deficient mice (Supplementary Fig. 5a, b). This apparent paradox could be explained by the lower force developed by DRP1-deficient muscles compared with control muscles and consequently, less ATP consumption during contraction. Thus, acute inhibition of DRP1 causes muscle loss and important weakness, but ameliorates fatigue.

### Acute deletion of *Drp1* causes mitochondrial dysfunction

**Fig. 5:**
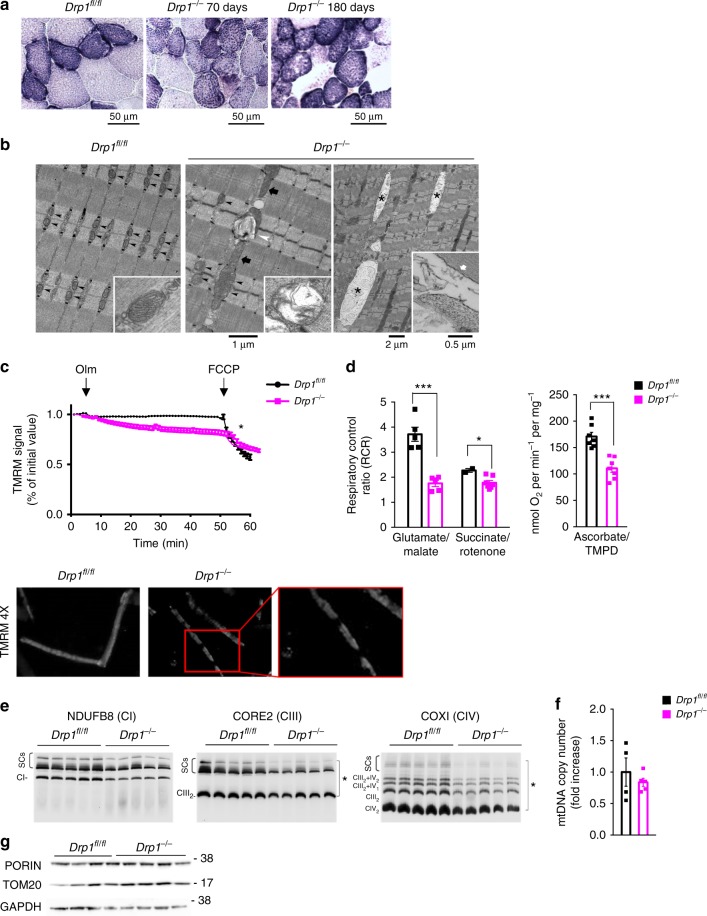
Ablation of DRP1 in adulthood modifies mitochondrial shape and reduces RCS assembly, Complex-I-, Complex-II-, and complex-IV-dependent respiration. **a** SDH staining indicating the presence of bigger mitochondria in KO muscles compared with control. **b** Representative electron micrographs of EDL muscles of controls and *Drp1*^−/−^. Left panel: in WT mitochondria (pointed by black arrowheads) are placed in proximity of Z lines and usually exhibit an electron-dense matrix (inset). Center panel: in *Drp1*^−/−^ mice mitochondria are often larger in size, oriented longitudinally (large arrows), and damaged (white arrowhead and inset). Right panel: a *Drp1*^−/−^ fiber presenting *spindle-shaped* regions (asterisks) which are surrounded by a membrane (inset, white arrow) and that contains vacuoles. **c** Absence of DRP1 induces mitochondrial depolarization. Isolated adult fibers were loaded with TMRM. Oligomycin and the protonophore FCCP were added at the indicated time points. TMRM staining was monitored in 18 fibers for WT, 52 fibers for KO. Lower panel: representative images of adult myofibers showing altered mitochondrial distribution in DRP1-null muscles. Myofibers were loaded with the potentiometric die TMRM. **d** Respiratory control ratio (RCR) of muscle isolated mitochondria energized with 5 mM/2.5 mM GLU/MAL or 2 mM rotenone/10 mM succinate or 3 mM ascorbate/10 mM TMPD. The data represent average ± SEM (GLUT/MAL, *n* = 5 each condition; succinate/rotenone: WT, *n* = 2; KO, *n* = 7; ascorbate/TMPD, *n* = 7 each condition). **e** The assembly of CIII and CIV in RCS is significantly reduced in mitochondria of DRP1-deficient muscles. Representative Blue Native PAGE analysis showing RCS developed and normalized for individual respiratory chain complexes. CI subunit NDUFB8 (left panel), CIII-Core2 protein 2 (middle panel), and CIV subunit COXI (right panel). **f** Mitochondrial DNA copy number quantification in controls and KO muscles. mtDNA was amplified by RT-PCR from the total DNA of gastrocnemius muscles of the indicated genotype. The data are normalized to controls and represent the average ± SEM (WT, *n* = 4; KO, *n* = 5). **g** Mitochondrial mass revealed by TOM20 and porin was not affected by DRP1 deletion (*n* = 4). Two-tailed unpaired Student’s *t* test was used. Statistical significance: *p ≤ 0.05; ***p ≤ 0.001

SDH staining was normal, but size of the puncta in oxidative fibers was increased, suggesting that mitochondria were bigger (Fig. 5a). Analyses of the mitochondrial ultrastructure by EM confirmed the presence of morphological modifications in *Drp1*^−/−^ fibers compared with controls. In *Drp1*^fl/fl^ EDL fibers (left panel of Fig. 5b), mitochondria are usually placed at the I band in proximity of Z lines (pointed by arrowheads), next to the calcium release units (CRUs)^30^. Healthy mitochondria usually exhibit an electron-dense matrix, with parallel internal cristae (Fig. 5b, inset). In *Drp1*^−/−^ fibers, about 10% of mitochondria are damaged (versus only 2% in WT), larger in the size (white arrowhead and inset in the center panel of Fig. 5b), and often longitudinally oriented between myofibrils (the center panel of Fig. 5b; large arrows). Moreover, the relative fiber volume occupied by the mitochondria is increased when compared with WT, even if their number is reduced (Supplementary Table 1). Finally, about 20% of fibers from *Drp1*^−/−^ muscles contains spindle-shaped structures, which are surrounded by a membrane and contains vacuoles, possibly representing degeneration of large swollen mitochondria (right panel of Fig. 5b, asterisks). Consistent with the morphological abnormalities, myofibers from tamoxifen-treated *Drp1*^−/−^ flexor digitorum brevis (FDB) muscle showed an increased incidence of the mitochondria that depolarized when the F_1_F_0_-ATPase was blocked by oligomycin^31^ (Fig. 5c). Moreover, TMRM staining revealed the area of the myofiber where mitochondria were completely depolarized (Fig. 5c). Accordingly, mitochondrial respiration (Fig. 5d; Supplementary Fig. 6a) was reduced, while complex activity is not greatly affected (Supplementary Fig. 6b). We also revealed a general reduction of the subunits of the different respiratory chain complexes, being complex-I the most affected (Supplementary Fig. 6c), and of supercomplex assembly (Fig. 5e; Supplementary Fig. 6d). Similar to the conditional knockout, the mitochondrial DNA content and mitochondrial mass revealed by TOM20 and porin did not differ between the two genotypes (Fig. 5f, g; Supplementary Fig. 6d). However, despite the alteration in respiration, mitochondrial ROS production was not increased by DRP1 ablation (Supplementary Fig. 6e). In conclusion, *Drp1* ablation in adult animals alters mitochondrial morphology and function independently of mtDNA content and mitochondrial mass.

### Acute deletion of *Drp1* induces ER stress and blocks autophagy

**Fig. 6:**
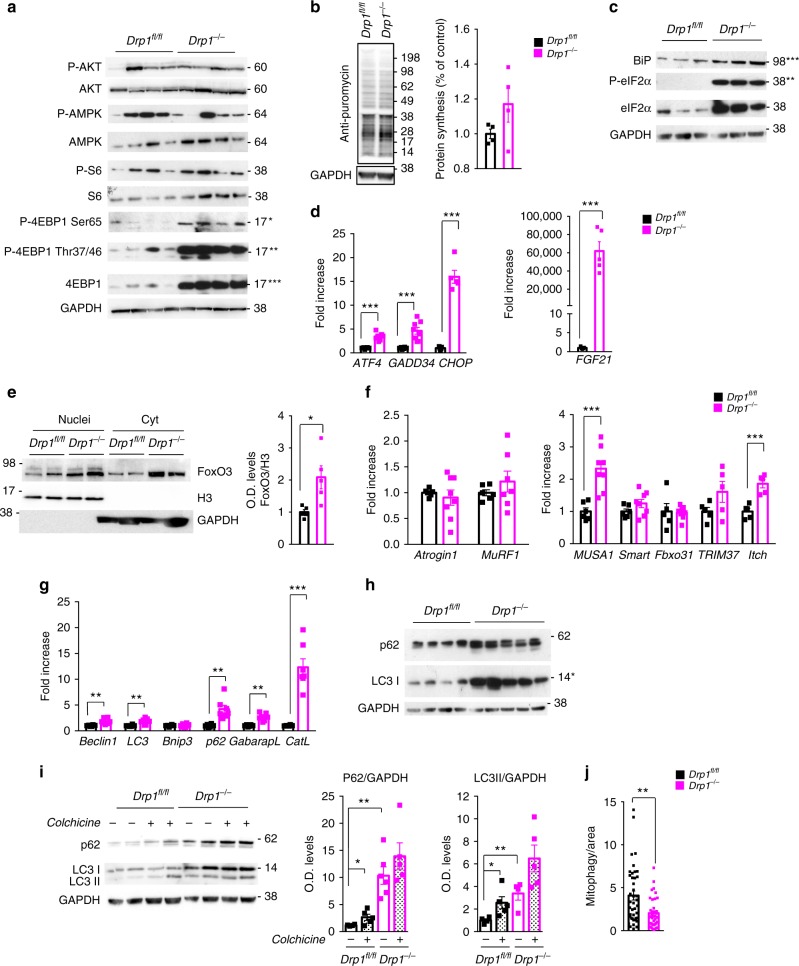
Protein degradation pathways are activated with acute inhibition of DRP1 in adult muscles. **a** The total protein extracts from adult muscles were immunoblotted with indicated antibodies. A representative immunoblot of four independent experiments is shown. Statistical significance, of specified densitometric ratios is indicated with asterisks on the right. **b** In vivo SUnSET technique shows no differences in protein synthesis rate during adulthood (*n* = 4). **c** Representative immunoblot (*n* = 4) and RT-PCR analysis (*n* ≥ 5) (**d**) showing the activation of the unfolded protein response and the upregulation of FGF21 in DRP1 KO muscles. **e** Immunoblot and densitometric quantification showing the accumulation of FoxO3 in the nuclear fraction of tibialis anterior muscles from *Drp1*^*−/*−^ mice (*n* = 5 each condition). **f** RT-PCR analysis of transcriptional levels of the muscle-specific ubiquitin ligases Atrogin1 and MuRF1 and the novel ubiquitin–ligases MUSA1, SMART, FbxO31, TRIM37, and Itch. The data represent average ± SEM (at least *n* = 5 per condition). **g** Quantitative PCR analysis of autophagy-related transcripts showing a significant induction of autophagy markers in Drp1^−/−^. **h** Western blot analysis of autophagy marker showing an impaired autophagy in *Drp1*^−/−^ (WT, *n* = 4; KO, *n* = 5). **i** Immunoblots and relative densitometric quantification from colchicine treated mice support a block in autophagic flux after Drp1 inhibition (*n* ≥ 5 mice per each condition). **j** Keima assay indicating the decreased mitophagy in DRP1-KO myofibers (WT, *n* = 41; KO, *n* = 39). Two-tailed unpaired Student’s *t* test was used. Statistical significance: **p ≤ 0.01; ***p ≤ 0.001

Similar to the results obtained in conditional *Drp1*-deficient mice, the Akt-mTOR axis appeared inconsistent with the atrophic phenotype being 4EBP1 robustly hyper-phosphorylated in two mTOR-dependent sites and S6 unchanged (Fig. 6a; Supplementary Fig. 7a). When we checked protein synthesis, in vivo, we did not find any difference (Fig. 6b; Supplementary Fig. 7b). Consistent with the conditional knockout, we found an activation of the UPR pathway. Bip/Grp78 and p-eIF2α (Fig. 6c; Supplementary Fig. 7a) and the transcripts of *ATF4* and downstream genes, including *FGF21*, were induced in DRP1-null muscles (Fig. 6d; Supplementary Fig. 7c). FGF21 blood levels were also increased of 20-fold in *DRP1* knockout mice when compared with controls (Supplementary Fig. 7d). *FoxO3* is the master regulator of the atrophy program and its activity is controlled at the transcriptional level and by multiple pathways via post-translational modifications, which include phosphorylation, acetylation, oxidation, and methylation^26,32^. Therefore, we monitored the presence of FoxO3 in the nuclear fraction as a readout of its activity and found an increased nuclear translocation in DRP1-deficient mice (Fig. 6e). The increase of the total 4EBP1 is also consistent with FoxO activation, being 4EBP1 a downstream target. Then we checked the status of the ubiquitin–proteasome and autophagy–lysosome systems. Interestingly, while *atrogin1* and *MuRF1* did not change, other two FoxO-dependent ubiquitin ligases *MUSA1*^28,33^ and *Itch* were upregulated in DRP1-deficient muscles (Fig. 6f). We then monitored the FoxO-dependent autophagy-related genes and we found an upregulation of most of these genes (Fig. 6g).

However, when we checked autophagosome formation, we did not detect an increase of lipidated LC3II, but instead we found an accumulation of LC3I, suggesting an autophagy impairment (Fig. 6h; Supplementary Fig. 7a). Next, we tested autophagy flux and we found no increase of p62 and LC3II upon colchicine treatment in DRP1-deficient mice (Fig. 6i). These findings confirm that autophagy is inhibited. Since mitochondrial fragmentation is blocked, we tested whether mitophagy is also impaired. Adult muscles were transfected with a pH-sensitive mitochondrial targeted probe that changes the fluorescence upon medium acidification (mitoKeima). Therefore, this probe reflects the autophagy-dependent delivery of the mitochondria to lysosome (mitophagy). The measurements confirmed that mitophagy is reduced in DRP1-deficient muscles (Fig. 6j). In conclusion, loss of DRP1 in adult muscle is sufficient to induce a catabolic condition that involves activation of the UPS system and inhibition of autophagy and mitophagy pathways^34^.

### Inhibition of DRP1 alters normal calcium homeostasis

**Fig. 7:**
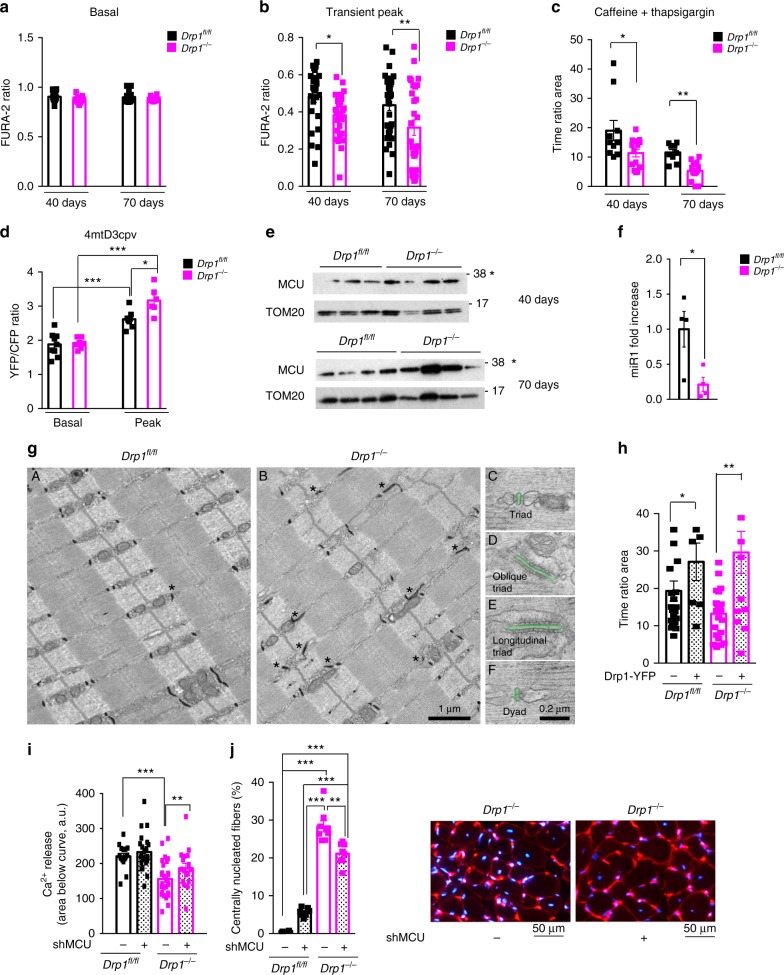
Acute DRP1 inhibition alters calcium homeostasis. **a** Cytosolic calcium level at rest is not affected in KO muscles (40 days: WT, *n* = 24; KO, *n* = 28; 70 days, *n* = 19 fibers). **b** Amplitude of cytosolic Ca^2+^ transients induced by 0.5 Hz electrical pulses is lower in *Drp1*^−/−^ mice (40 days, WT, *n* = 25; KO, *n* = 28 fibers; 70 days, WT, *n* = 33; KO, *n* = 29 fibers). **c** Cytosolic calcium transients induced by caffeine–thapsigargin are strongly reduced (40 days, WT, *n* = 10; KO, *n* = 14 fibers; 70 days, WT, *n* = 13 fibers; KO, *n* = 14 fibers). **d** Mitochondrial matrix-free calcium concentration is not affected at rest but is higher in *Drp1*^−/−^ compared with control during a train of electrical stimulation at 60 Hz, 2 s duration (WT, *n* = 9; KO, *n* = 7 fibers; Peak, *n* = 6 each condition). **e** Immunoblot analysis of isolated mitochondria. MCU levels were normalized to TOM20 levels. **f** Quantitative RT-PCR analysis showing a significant decrease of miR1 levels in *Drp1*^−*/*−^ skeletal muscle (*n* = 4 per condition). The data represent average ± SEM. **g** Ultrastructure and geometry of CRUs in DRP1 KO muscles. Inserts A and B show T-tubule staining in WT (**A**) and in DRP1 KO (**B**); the frequency of oblique/longitudinal tubules increases (stars) when compared with control samples. Inserts **C**–**F** are representative EM pictures showing the structural geometry of a normal, an oblique, a longitudinal triad and of a dyad respectively. In inserts **A** and **B**, T-tubules are stained in black while in inserts C–F, T-tubules has been false-labeled in green. **h** Cytosolic calcium transients induced by caffeine–thapsigargin after the reintroduction of DRP1 (Drp1-YFP) in muscle fibers. Cytosolic calcium transients in *Drp1*^−*/−*^ in the presence of Drp1-YFP show no difference compared with control fibers (*Drp1*^*fl/fl*^, *n* = 22; *Drp1*^*fl/fl*^—Drp1-YFP, *n* = 7; *Drp1*^*−/*−^, *n* = 22; *Drp1*^−*/*−^ – Drp1-YFP, *n* = 13). **i** Inhibition of mitochondria Ca^2+^ uptake via MCU knockdown restored cytosolic Ca^2+^ levels during caffeine–thapsigargin treatment (WT, *n* = 17 fibers; WT shMCU, *n* = 23 fibers; KO, *n* = 27 fibers; KO shMCU, *n* = 17 fibers). **j** MCU inhibition reduces centrally nucleated fibers in KO muscles. For all experiments a minimum of three mice was used for each condition. Two-tailed unpaired Student’s *t* test and two-way analysis of variant (ANOVA) were used. *p ≤ 0.05; **p ≤ 0.01; ***p ≤ 0.001

Acute deletion of DRP1 causes weakness, myofiber degeneration, ER stress, and appearance of the swollen/dysfunctional mitochondria. All these features can be triggered by disruption of Ca^2+^ homeostasis. Therefore, we initially measured cytosolic calcium of *Drp1*^−/−^ and controls in basal condition and found no difference (Fig. 7a). However, when we stimulated the Ca^2+^ release from the sarcoplasmic reticulum (SR), either by electrical stimulation or by caffeine/thapsigargin treatment, we showed a reduction of the cytosolic Ca^2+^ transient in DRP1-deficient myofibers (Fig. 7b, c; Supplementary Fig. 8a, b). These changes occurred early after 40 days from tamoxifen treatment when animals have not yet lost muscle mass and progressively increased at 70 days.

To test whether Ca^2+^ store in SR was reduced, we checked the concentration of this ion by transfecting adult muscle with SR-targeted cameleon probes. However, no differences were found between *DRP1* knockout and controls, either in basal or after electrical stimulation (Supplementary Fig. 8c, d, g). Importantly, the changes of Ca^2+^concentration in the SR after electrical stimulation did not differ between controls and DRP1-deficient mice, suggesting that the two genotypes release the same amount of Ca^2+^ (Supplementary Fig. 8g). Consistently, calsequestrin, the most important Ca^2+^ buffering protein in the SR, did not change between knockout and controls (Supplementary Fig. 9c). We then measured free Ca^2+^concentration in the mitochondrial matrix by using a specific cameleon probe and we found that the peak of Ca^2+^ concentration reached during high-frequency electrical stimulation was higher in DRP1-deficient myofibers (Fig. 7d; Supplementary Fig. 8e, f). This suggests that the presence of an active and not depolarized mitochondrial population was able to uptake Ca^2+^ from the cytosol.

Importantly, when we checked the level of the mitochondrial calcium uniporter (MCU), the mitochondrial channel for Ca^2+^ uptake, we showed an increase of MCU in mitochondria of DRP1-deficient mice (Fig. 7e; Supplementary Fig. 9a). This increase is not consequent to transcription because the *MCU* transcripts did not differ between control and DRP1 knockout (Supplementary Fig. 9b). We have recently shown that MCU translation is controlled by miR1^35^. When we monitored miR1 expression, we found that it was downregulated in DRP1-deficient muscles when compared with controls (Fig. 7f). Therefore, DRP1 inhibition causes increased MCU expression via miR1 downregulation and enhanced Ca^2+^ uptake of the mitochondria.

The reduction of cytosolic Ca^2+^ upon stimulation could, in principle, also result from modifications of CRUs, the structures deputed to excitation contraction (EC) coupling. To address this point, we performed EM analyses and found some modification in CRUs compared with WT fibers (Fig. 7g). Indeed, we showed an increased frequency of oblique/longitudinal triads (inserts D and E) and a decrease in the number/area of CRUs compared with WT fibers (Fig. 7g, inserts D–F; Supplementary Table 2, column A). The combined reduction in the number of the mitochondria (Supplementary Table 1, column B) and of CRUs (Supplementary Table 2, column A), results in a reduced number of mitochondria-CRUs pairs (Supplementary Table 2, column D). When we checked the expression level of the SR-mitochondrial tethering protein Mitofusin2 and the ER chaperone, calnexin^36^, we found a downregulation in their expression (Supplementary Fig. 9c). Thus, the impairment of the SR–mitochondria tethering and ER chaperon proteins would explain the abnormalities of CRUs and the presence of ER stress^36,37^.

### DRP1 rescue or MCU inhibition normalizes Ca^2+^ homeostasis

To test the hypothesis that the DRP1-dependent mitochondrial shape is relevant for Ca^2+^ homeostasis alteration, we performed a rescue experiment. Re-expressing DRP1 protein in DRP1-deficient mice enhanced the wave of cytosolic Ca^2+^ induced by caffeine stimulation. Interestingly, DRP1-mediated mitochondrial fragmentation in control animals also increased the raise of cytosolic Ca^2+^ induced by caffeine/thapsigargin, supporting the hypothesis that mitochondrial dynamic impacts on Ca^2+^ homeostasis (Fig. 7h). Importantly, DRP1 overexpression reverted or induced UPR in DRP1 knockout and control mice, respectively (Supplementary Fig. 9d). Finally, to prove that abnormal mitochondrial Ca^2+^ homeostasis contributes to the phenotype of DRP1 knockout, we knocked down *MCU* in mice. FDB muscles were transfected by electroporation with expression vectors that encode shRNAs against *MCU*, and 14 days later muscles were collected, dissected, and analyzed with Ca^2+^ probe. Inhibition of *MCU* normalized the amplitude of the wave of cytosolic Ca^2+^ induced by caffeine stimulation in *Drp1*^−*/−*^ (Fig. 7i). Next, we asked whether by preventing mitochondrial Ca^2+^ uptake we could ameliorate myofiber survival and ER stress. Muscles were transfected by AAVs with expression vectors for oligos against *MCU*, as previously described^38^. By reducing MCU expression for two months, we reduced the amount of center-nucleated fibers (Fig. 7j) and suppressed UPR (Supplementary Fig. 9e). Altogether, these findings confirm that inhibition of the fission machinery alters the SR–mitochondria tethering, decreases CRU units, increases mitochondrial Ca^2+^ uptake that, altogether, reduces Ca^2+^ availability for contraction, and simultaneously induces myofiber death, due to mitochondrial Ca^2+^ overload.

## Discussion

**Fig. 8:**
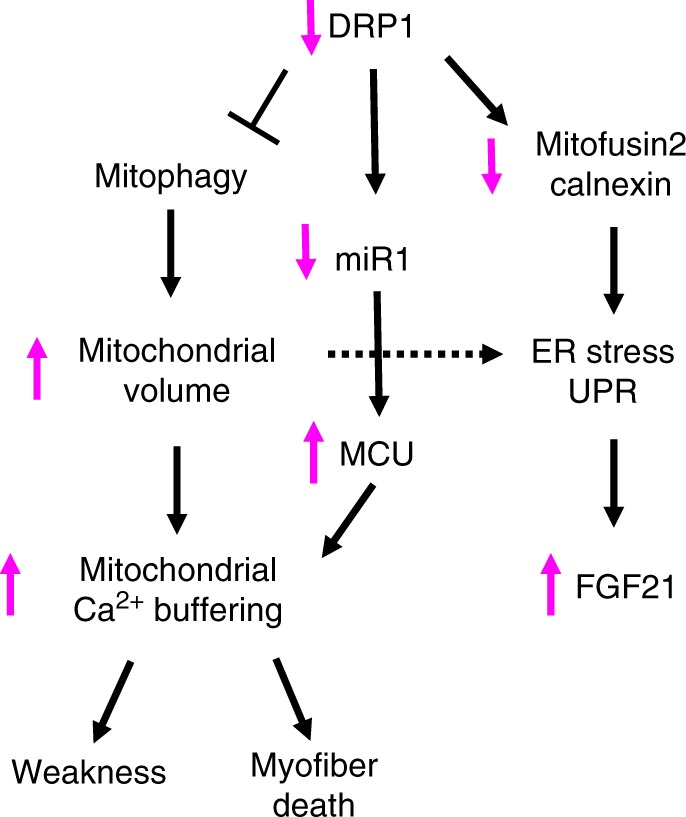
Scheme of the mechanisms induced by acute DRP1 inhibition

Our study provides insights into the in vivo functions of DRP1 in the muscle of mammals, and therefore sheds light on why DRP1 inhibition causes myopathy in different inherited and acquired diseases^13–15,17–20^. The finding that two different knockouts mice, muscle-specific and inducible muscle-specific, result in striking phenotypes (Supplementary Table 3) underlines the critical role of DRP1 in mitochondria function and muscle homeostasis. Like other systems, such as autophagy, both chronic inhibition or acute hyperactivation of mitochondrial fission result in detrimental effects on muscle mass and function, but with different phenotypes and mechanisms. In fact, we and others have shown that overexpression of DRP1 is sufficient to activate an atrophy program via the AMPK-FoxO3 axis^10,11^. However, while the initial outcome of genetic DRP1 ablation is identical to its overexpression, i.e., muscle atrophy via FoxO, our loss-of-function study also reveals the appearance of center-nucleated fibers, a sign of myopathy, and the involvement of different signaling pathways, including MCU upregulation, ER-stress, UPR activation, and great FGF21 induction (Fig. 8). Another peculiarity of the loss-of-function approach is the decrease of the ER-mitochondria tethering protein mitofusin2 and the down- and upregulation of the ER chaperones calnexin and Bip/Grp78, respectively^11^. Both the decreased mitofusin2 and calnexin expression explain why ER stress and UPR are induced by DRP1 inhibition^36,37^. ER stress and UPR activation are well-known triggers of FGF21 production in different organs, such as the liver, pancreas, or adipose tissue^39^. Our data show that muscles can be an important source of blood FGF21, when mitochondria are dysfunctional, irrespective of whether this occurs because of a specific mitochondrial dysfunction (as reported here and in ref. ^13^) or because of mitochondrial dysfunction secondary, for example, to autophagy impairment^40^. The increase of FGF21 can explain the observed metabolic changes, such as basal hypoglycemia, liver GH resistance, and the reduced animal size of conditional knockout mice. However, different phenotypes have been described in mice with high level of FGF21 expression. For instance, FGF21 induction after OPA1 ablation caused systemic inflammatory response and precocious senescence of epithelial tissues such as skin, liver, and gut that resulted in premature death^13^. In DRP1 knockout mice, the main phenotype is not related to ageing but to muscle degeneration and regeneration. The absence of senescence despite the FGF21 induction in DRP1 knockout mice may be explained by the lower level of FGF21 in the serum of DRP1 knockout when compared with OPA1^−/−^ mice. Alternatively, FGF21 needs to synergize with other factors to elicit the pro-ageing action. Acute deletion of OPA1 in muscles triggers an important IL6 and IL1 upregulation. This induction of inflammatory cytokines is ROS mediated^13^. Interestingly, the inflammatory response is not present and oxidative stress is not increased in DRP1 knockout mice (Supplementary Figs. 9f, 6e). Therefore, the presence of high FGF21 levels associated with high inflammatory cytokines in blood is peculiar of OPA1 knockout mice and may explain the different phenotypes of these inducible muscle-specific knockout mice.

The main peculiarity of DRP1 knockout is an abnormal Ca^2+^ homeostasis, which is not present in OPA1-deficient mice. These changes may result from modifications of the EC-coupling machinery (Fig. 7g; Supplementary Table 2) but in principle also from the the mitochondria (Figs. 5b, 7d; Supplementary Table 1), which being placed in close proximity to sites of Ca^2+^ release^41^ are the first to sense the Ca^2+^ transient via MCU^42,43^. Importantly, this Ca^2+^ dysregulation happens before muscle wasting starts, suggesting that it may have a pathogenetic role in DRP1 KO phenotype. The concept that mitochondria affect cytosolic Ca^2+^ availability is still debated. In fact, the contribution of the mitochondria to cytosolic Ca^2+^ homeostasis is believed to be dispensable in physiological condition. However, our present data show that the expression of MCU is increased when DRP1 is acutely deleted (Fig. 7e), and that intracellular Ca^2+^ levels are normalized when MCU was knocked down in vivo (Fig. 7i). Moreover, experiments of DRP1 rescue in knockout and overexpression in control mice (Fig. 7h) support the concept that the mitochondrial network may impact on Ca^2+^ homeostasis. The increased expression of MCU in *Drp1*^*−/*−^ fibers could induce mitochondrial Ca^2+^ overload, and possibly explains the increased presence of the swollen and damaged mitochondria (Fig. 5 b; Supplementary Table 1). In fact, it is well known that Ca^2+^ overload triggers permeability transition pore (PTP) opening and mitochondrial swelling^44^. The swelling of mitochondria could, in turn, induce the release of death factors such as endoG, AIF, and cytochrome c that per se, or in combination with other factors, may cause myofiber death in DRP1-deficient muscles. In fact, when we knocked down MCU, we preserved myofiber survival revealed by the decrease of center-nucleated fibers. We did not completely block the degeneration/regeneration process because the efficiency of transfection was low, and consequently, MCU was reduced only in some myofibers. Interestingly, this scenario is very similar to what happens in Duchenne muscle dystrophy, in which membrane leakage leads to Ca^2+^ entrance and mitochondria swelling. Indeed, it has been shown that inhibition of PTP opening is beneficial in dystrophic muscles^45–47^.

An apparent surprising finding is the improvement of fatigue in DRP1-deficient mice, despite the presence of a mitochondrial dysfunction. It is important to note that fatigue was measured before the appearance of degenerating/regenerating events. The apparent paradox could be explained by the lower force developed by DRP1-deficient muscles compared with control muscles. The reduction of force due to less contractile proteins and of cytosolic Ca^2+^ level leads to a decreased ATP consumption during contraction by myosin and SERCA ATPase pump, delaying ATP drop and consequently, improving fatigue. Alternative explanations are also possible: (i) the fiber type switch toward 2X fiber may contribute to fatigue improvement; (ii) the increase of mitochondrial Ca2+ uptake, in a stage that is not deleterious for myofiber survival, stimulates TCA cycle that helps energy production during repetitive tetanic contraction; (iii) the presence of a hyperfused mitochondrial network compensates the mitochondrial dysfunction due to DRP1 inhibition and preserves ATP production during multiple tetanus; (iv) because DRP1-deficient muscles show less oxidative stress, myofibrillar proteins are better preserved from ROS-mediated damage during repetitive contraction.

In conclusion, even though both fusion and fission machinery inhibitions induce mitochondrial dysfunction, revealed by decreased respiration, supercomplex formation, and mitochondrial membrane potential, the signaling pathways and the biological effects downstream mitochondria are peculiar for fusion or fission deficiency. This finding opens the field to a novel concept in which the shape and spatial organization of mitochondrial network, more than mitochondrial respiration, are sensed by the cell and activate a peculiar biological response. Therefore, the shape of the mitochondria is critical for mitochondrial function, but triggers different signaling pathways and biological processes that are peculiar of fusion or fission impairment.

Finally, therapeutic approaches that maintain a healthy and good shaped mitochondrial network should be considered for the inherited and acquired disorders, characterized by both muscle wasting and mitochondrial dysfunction.

## Methods

### Handling and generation of muscle-specific *Drp1* null mice

Animals were handled by specialized personnel under the control of inspectors of the Veterinary Service of the Local Sanitary Service (ASL 16 - Padova), the local officers of the Ministry of Health. The use of the animals and the experimental protocol was approved by the ethical committee and by the animal welfare coordinator of the OPBA, University of Padova All procedures are specified in the projects approved by the Italian Ministero Salute, Ufficio VI (authorization numbers 1060/2015 PR), and were conducted in accordance with the relevant codes of practice for the care and use of animals for scientific purposes. Muscles were removed at various time periods and frozen in liquid nitrogen for subsequent analyses.

To generate constitutive muscle-specific *Drp1* knockout animals, mice bearing *Drp1* floxed alleles (*Drp1*^fl/fl^) were crossed with transgenic mice expressing Cre under the control of a Myosin Light Chain 1 fast promoter (MLC1f-Cre)^21^. Experiments were performed on newborns at postnatal day 12. Cre-negative littermates were used as controls.

A second knockout model with inducible muscle-specific deletion of *Drp1* was obtained by crossing the *Drp1*^fl/fl^ line with mice carrying Cre-ER driven by human skeletal actin promoter (HSA). Tamoxifen-induced Cre LoxP recombination was activated by oral administration of tamoxifen-containing chow (Tam400/Cre ER Harlan), which was administered ad libitum for 5 weeks. Muscles were collected 5 weeks after the tamoxifen diet finished. Cre-negative littermates, also receiving tamoxifen treatment, were used as controls. Adult mice (3- to 5-month-old) of the same sex and age were used for each individual experiment. For fasting experiments, control animals were fed ad libitum; food pellets were removed from the cages of the fasted animals for 24 h before killing and muscles collection. PCR genotyping was performed with the following primers:

DRP1 Fw: CAGCTGCACTGGCTTCATGACTC

DRP1 Rv: GTCAACTTGCCATAAACCAGAG

Cre Fw: CACCAGCCAGCTATCAACTCG

Cre Rv: TTACATTGGTCCAGCCACCAG

### Gene-expression analyses

The total RNA was prepared from gastrocnemius muscles or liver using TRIzol (Invitrogen). Complementary DNA was generated from 0.4 μg of RNA reverse-transcribed with SuperScript III Reverse Transcriptase (Invitrogen). Duplicates of cDNA samples were then amplified on the 7900HT Fast Real-Time PCR System (Applied Biosystems) using the Power SYBR Green RT-PCR kit (Applied Biosystems). All data were normalized to *GAPDH* expression and plotted in arbitrary units as mean ± SEM. The oligonucleotide primers used are shown in Supplementary Table 4.

*IGF1* quantification was performed using TaqMan® Universal PCR Master Mix and the specific TaqMan primers *IGF1 class II* (Mm 00439559_m1, Life Technologies). The data were normalized to *GAPDH* expression (Mm 99999915_g1, Life Technologies). miR1 was quantified using single specific RT-qPCR using TaqMan MicroRNA Assays (Applied Biosystems by Thermo Fisher Scientific, Waltham, MA, USA). cDNA synthesis was obtained starting from 5 µl of RNA with the TaqMan MicroRNA Reverse Transcription Kit, and the amplification was subsequently performed with TaqMan Fast Universal PCR Master Mix using 1 µl of cDNA and specific TaqMan primers (miR1 Assay ID 002222). The results are expressed as mean ± SEM.

### Immunoblotting

Muscles were powdered by pestle and mortar and lysed in a buffer containing 50 mM Tris pH 7.5, 150 mM NaCl, 5 mM MgCl_2_, 1 mM DTT, 10% glycerol, 2% SDS, 1% Triton X-100, Roche Complete Protease Inhibitor Cocktail, 1 mM PMSF, 1 mM NaVO3, 5 mM NaF, and 3 mM b-glycerophosphate. The lysis buffer used for MEF and C2C12 cells contained 50 mM Tris pH 7.5, 150 mM NaCl, 5 mM MgCl_2_, 1 mM DTT, 10% glycerol, 1 mM EDTA, 0.5% Triton X-100, and the protease inhibitors listed above. Samples were immunoblotted and visualized with Super-Signal West Pico Chemiluminescent substrate (Pierce). Blots were stripped using Restore Western Blotting Stripping Buffer (Pierce) according to the manufacturer’s instructions and reprobed if necessary. List of antibodies is depicted in Supplementary Table 5.

Uncropped blots are shown in Supplementary Fig. 10.

### Nuclear protein extraction

Tibialis anterior muscles were lysed in 700 μl of lysis buffer (10 mM HEPES, 10 mM KCl, 5 mM MgCl_2_, 0.5 mM DTT, 1 mM PMSF, 1 mM sodium vanadate, 50 mM sodium fluoride, and 25 mM β-glycerophosphate) and then centrifuged to separate nuclear fraction from cytosolic fraction. Proteins from nuclear fraction were obtained after incubation for 10 min at 70 °C, 46 g, and centrifuged for 10 min, 15.871 g. Proteins from the cytosolic fraction were precipitated overnight after the addition of cold acetone and centrifuged for 5 min at 12,000 *g*.

### Imaging and transmission EM

Cryosections of both P12 newborns hindlimb cross-sections and adult TA were stained for H&E and SDH. The total myofiber numbers and CSA in conditional model were calculated from entire hindlimb cross-section based on assembled mosaic image (×20 magnification). Adult mice CSA was performed on TA as described^29^. For EM, EDL muscles were dissected from killed animals, pinned on a Sylgard dish, fixed at room temperature with 3.5% glutaraldehyde in 0.1 M sodium cacodylate buffer (NaCaCO) (pH 7.4), and stored in the fixative at 4 °C. Fixed muscles were then postfixed in a mixture of 2% OsO_4_ and 0.8% K_3_Fe(CN)_6_ for 1–2 h, rinsed with 0.1 M NaCaCO with 75 mM CaCl_2,_ en-block stained with saturated uranyl acetate, and embedded for EM in epoxy resin (Epon 812) as in ref. ^48^. Ultrathin sections (~40 nm) were cut in a Leica Ultracut R microtome (Leica Microsystem, Austria) using a Diatome diamond knife (DiatomeLtd. CH-2501 Biel, Switzerland) and examined at 60 kV after double-staining with uranyl acetate and lead citrate, with a FP 505 Morgagni Series 268D electron microscope (FEI Company, Brno, Czech Republic), equipped with Megaview III digital camera (Munster, Germany) and Soft Imaging System (Germany).

### Quantitative analyses of the mitochondria

The data contained in Supplementary Table 1 and Supplementary Table 2 were collected from 5 to 6-month-old wild-type (WT, *n* = 3 mice) and Drp1-KO (*n* = 4 mice) EDL muscles. Micrographs of non-overlapping regions were randomly collected from transversal and longitudinal sections of internal fiber areas of EDL muscles. Sample size is detailed in the Supplementary Table legend.

Mitochondrial volume (Supplementary Table 1, column A and Supplementary Fig. 2a) was determined using the well-established stereology point-counting technique^49,50^ in micrographs taken from transversal sections at ×7.100 magnifications. Briefly, after superimposing an orthogonal array of dots at a spacing of 0.20 μm to the electron micrographs, the ratio between numbers of dots falling within mitochondrial profiles and the total number of dots covering the whole image was used to calculate the relative fiber volume occupied by the mitochondria.

The number of the mitochondria and CRUs/area (Supplementary Table 1, column B; Supplementary Table 2 column A) was evaluated in micrographs taken at ×14.000 magnifications in longitudinal sections and reported as average number over 100 μm^2^. If an individual mitochondrion extended from one I band to another, it was counted in both. In each EM image, we also determined (i) orientation (oblique/longitudinal triads) of CRUs; (ii) morphology (dyads) of CRUs expressed as percentages over the total number of CRUs; (iii) the number of mitochondria-CRUs pairs/area (Supplementary Table 2, columns B, C, D, respectively).

The number of severely damaged mitochondria (Supplementary Table 1, column C) was evaluated in micrographs of longitudinal sections taken at ×14.000 magnifications; their number is reported as average number over 100 μm^2^ and as the percentage of the total number (in parenthesis). Mitochondria with any or several of the following ultrastructural alterations were classified as severely damaged: (a) presenting disruption of the external membrane; (b) presence of internal vacuolization and/or disrupted internal cristae; and (c) containing *myelin-figures*.

The average area of the mitochondria (Supplementary Table 1, column D and Supplementary Fig. 2a) was measured in the same set of micrographs using the Soft Imaging System (Germany) provided with the electron microscope. Only mitochondria which were entirely visualized in the micrograph were measured.

### Measurement of mitochondrial DNA copy number

The total gastrocnemius DNA was isolated using Puregene Cell and Tissue Kit (Qiagen) and was amplified using specific primers for mtCOXII and 18S by real-time PCR using the Power SYBR Green RT-PCR kit (Applied Biosystems). The mtDNA copy number was calculated using 18S amplification as a reference for nuclear DNA content. Real-time PCR was performed with the following primers:

18S Fw: CATTCGAACGTCTGCCCTATCA

18S Rw: GGGTCGGGAGTGGGTAATTTG

COXII Fw: GCCGACTAAATCAAGCAACA

COXII Rw: CAATGGGCATAAAGCTATGG

### Mitochondrial assays

Muscle mitochondria from the indicated genotype were isolated by differential centrifugation steps^51^. To detect RCS, Blue Native PAGE was performed by resupending mitochondrial pellets in Native Buffer (Invitrogen) plus 4% Digitonin (SIGMA) to a final concentration of 10 μg/µl and incubated for 1 h on ice. After 20 min of centrifugation at 16,000 *g*, the supernatant was collected and one-third of digitonin percentage of sample buffer 5% G250 (Invitrogen) was added. Then 50 μg of mitochondrial membrane proteins were loaded and run on a 3–12% Bis-Tris gel (Invitrogen), as described in the NativePAGE™ Novex® Bis-Tris Gel System manual. Western blot was performed using a Trans-Blot transfer cell (Bio-Rad) at 4 °C overnight.

To measure respiration, mitochondria (1 mg/ml) were incubated in experimental buffer (EB: 150 mM KCl, 10 mM Tris Mops, 10 mM EGTA-Tris, and 10 mM ATP). When indicated, mitochondria were transferred into a Clark’s type oxygen electrode chamber and 5 mM glutamate/2.5 mM malate or 2 mM rotenone/10 mM succinate or 3 mM ascorbate/10 mM TMPD were added. Basal O_2_ consumption was recorded (state 2), and after 2 min 100 mM ADP was added (state 3), followed by 2.5 mg/ml oligomycin (state 4) and 200 mM FCCP (state 3).

Assesment of mitochondrial respiratory chain enzymatic activities on muscles was determined spectrophotometrically^52^. The results were normalized to citrate synthase enzymatic activity.

### Mitochondria oxidative stress measurements

Mitochondrial targeted HyPer (Hydrogen Peroxide sensor) was used as an indicator of mitochondrial redox status. Briefly, adult FDB muscles were transfected by electroporation with mt-HyPer plasmid. After 7 days of transfection, single-muscle fibers were isolated from the control and knockout mice. Mt-HyPer fluorescence (excitation: 420 and 488 nm, emission: 515 nm) was measured for 8 min every 10 s and upon treatment with H_2_O_2_. The ratio of fluorescence intensities was determined by ImageJ Software.

### Mitochondrial membrane potential determination

Mitochondrial membrane potential was measured in isolated fibers from flexor digitorum brevis (FDB) muscles, as previously described^29,53^. Briefly, FDB myofibers were placed in 1 ml Tyrode’s buffer and loaded with 2.5 nM TMRM (Molecular Probes) supplemented with 1 µM cyclosporine H (a P-glycoprotein inhibitor) for 30 min at 37 °C. Myofibers were then observed at a Olympus IMT-2 inverted microscope (Melville, NY) equipped with a CellR imaging system. Sequential images of TMRM fluorescence were acquired every 60 s with a 20 × 0.5 UPLANSL N A objective (Olympus). At the times indicated by arrows, oligomycin (Olm, 5 µM) (Sigma) or the protonophore carbonyl cyanide p-trifluoromethoxyphenylhydrazone (FCCP, 4 µM) (Sigma) was added to the cell culture medium. We used oligomycin to inhibit ATP-synthase, because ATP-synthase can reversely transport protons across the inner mitochondrial membrane, so maintaining the potential also in dysfunctional mitochondria. For this reason, only this treatment allows us to detect real dysfunctional mitochondria that would inevitably dissipate the potential, losing TMRM signal. Images were acquired, stored, and then analysis of TMRM fluorescence over mitochondrial regions of interest was performed using ImageJ software (http://rsb.info.nih.gov/ij/).

### Free calcium concentration in the cytosol

FDB fibers were dissociated with collagenase were loaded with 5 μM Fura-2 AM (Molecular Probes, Invitrogen) in incubation buffer (125 mM NaCl, 5 mM KCl, 1 mM MgSO_4_,1 Mm KH_2_PO_4_, 5.5 mM glucose, 1 mM CaCl_2_, 20 Mm HEPES, and 1% bovine serum albumin, pH adjusted to 7.4 with NaOH), washed twice for 10 min with incubation buffer without BSA at 37 °C to retain the indicator in the cytosol, and immersed in imaging buffer (125 mM NaCl, 5 mM KCl, 1 mM CaCl_2_, 1 mM MgSO_4_, 1 mM KH_2_PO_4_, 5 mM glucose, 20 mM HEPES). After a minimum of 30 min, calcium signals were recorded using a dual-beam excitation fluorescence photometry setup (IonoOptix Corp.) at 25 °C. Fibers were electrically or chemically stimulated following the protocol described below. Ca^2+^ measurements were expressed as fluorescence ratio of the emission at 480 nm with reference to the excitation wavelengths of 360 and 380 nm, respectively.

To study calcium release under physiological condition of contractile activity, fibers were treated with BTS 20 μM to block spontaneous contraction, then electrically stimulated with trains of pulses at 0.5 Hz, followed by stimulation trains at higher frequency (60 Hz, 2 s).

Chemical release of calcium from the SR was induced by sequential administration of caffeine and thapsigargin. Caffeine 20 mM stimulates Ca^2+^ release from the SR through its action on RyR. Simultaneously, fibers were treated with thapsigargin 1 μM, an inhibitor of the SR/ER Ca^2+^ ATPase (SERCA).

Calibration of the Fura-2 AM signals and calculation of the free cytosolic calcium concentration: muscle fiber fluorescence R was converted to free cytosolic calcium concentration [Ca^2+^]CY using the equation [Ca^2+^]CY = (R − R_min_)/(R_max_ − R)·KD·β, where R is the ratio of fluorescence excited at 340 nm to that excited at 380 nm and KD is the affinity constant of Fura-2 for calcium, which was taken at 145 nM (Molecular Probes). R_min_ and R_max_ were determined in iono-permeabilized myofibers: R_min_ was the minimum R value measured in calcium-free solution, and R_max_ was the maximal R value measured in 2 mM calcium solution. β was derived as the F380 calcium-free/F380 calcium-saturated R. Under these assumptions, the resting cytosolic calcium concentration will be 71 nM at R = 0.87 and 87 nM at R = 0.92.

### Detection of free Ca^2+^ in the SR and mitochondrial matrix

Free Ca^2+^ concentrations in the mitochondrial matrix and SR lumen were determined using cameleon Ca^2+^ probes, 4mtD3cpv^54^ for mitochondria and D1-ER ^55^ for sarcoplasmic reticulum, kindly donated by Dr. R.Y. Tsien (University of California, San Diego).

The probes were expressed in FDB muscles by electroporation of plasmid vectors as described in the In vivo FDB electroporation section.

Mitochondrial and the SR-free Ca^2+^ levels were determined from the YFP/CFP ratio of the emitted lights of the cameleon probes excited with UV light in an inverted fluorescence microscope (Eclipse-Ti; Nikon Instruments). Fibers were placed in chamber containing imaging buffer on the movable stage of the microscope, and temperature was set at 25 °C. CFP fluorophores were excited by means of a Hg arc lamp using a 435-nm filter. CFP and YFP intensities were recorded by means of a cooled CCD camera (C9100-13; Hamamatsu), equipped with a dichroic mirror, at 535 nm (YFP emission) and 480 nm (CFP emission), respectively. Two images of 256 × 128 pixels each, corresponding, respectively, to YFP and CFP light emissions, were collected with a time resolution of 9 ms. YFP and CFP intensities were corrected for background and the ratio R was defined as follows: R = (YFP_fiber _− YFP_background_)/(CFP_fiber _− CFP_background_).

Electrically induced release of calcium from the SR was achieved by electrical stimulation, as reported above (trains of 2 s at 60 Hz). Chemical release of Ca^2+^ from the SR was induced by sequential administration of caffeine and thapsigargin. Caffeine 20 mM stimulates Ca2+ release from SR through its action on RyR. Simultaneously, fibers were treated with thapsigargin 1 μM, an inhibitor of the sarco/endoplasmic reticulum Ca2+ ATPase (SERCA).

### Cytosolic Ca^2+^ real-time imaging in MCU knocked down fibers

For real-time imaging, FDB muscles were digested in collagenase A (4 mg/ml) (Roche) dissolved in Tyrode’s salt solution (pH 7.4) (Sigma-Aldrich) containing 10% FBS (Thermo Fisher Scientific). Single fibers were isolated, plated on laminin-coated glass coverslips, and cultured in the DMEM with HEPES (Thermo Fisher Scientific), supplemented with 10% FBS. Fibers were maintained in culture at 37 °C with 5% CO_2_.

During the experiments, myofibers were maintained in Krebs–Ringer modified buffer (135 mM NaCl, 5 mM KCl, 1 mM MgCl_2_, 20 mM HEPES, 1 mM MgSO_4_, 0.4 mM KH_2_PO_4_, 5.5 mM glucose, pH 7.4) in the presence of 500 μM EGTA and 75 μM BTS (Tocris). In total, 20 mM caffeine and 1 μM thapsigargin (Sigma-Aldrich) were added when indicated to elicit Ca^2+^ release from intracellular stores with no consequent reuptake. Experiments were performed on a Zeiss Axiovert 200 microscope equipped with a 40×/1.3N.A. PlanFluor objective. Excitation was performed with a DeltaRAM V high-speed monochromator (Photon Technology International) equipped with a 75 W xenon arc lamp. Images were captured with a high-sensitivity Evolve 512 Delta EMCCD (Photometrics). The system is controlled by MetaMorph 7.5 (Molecular Devices) and was assembled by Crisel Instruments. FURA2-AM was excited every second at 340 and 380 nm, respectively, and images were acquired through a 515/20 bandpass emission filter (Chroma). Exposure time was set to 50 ms. Acquisition was performed at binning 1 with 200 of EM gain. Image analysis was performed with Fiji distribution of the ImageJ software^56^. Images were background subtracted, and ΔR/R_0_ was calculated. The data are expressed as area below curve integrating 150 s after stimulation.

### Mito-mKeima mitophagy assay

Mitochondria-targeted mKeima plasmid (mt-Keima) (MBL International) was used to monitor mitophagy in transfected FDB single fibers. mt-Keima is a coral-derived protein that exhibits both pH-dependent excitation and resistance to lysosomal proteases. These properties allow rapid determinations to whether the protein is in mitochondria or the in lysosome^57,58^. In fluorescence microscopy, ionized Keima is detected as a red fluorescent signal at acidic pH (lysosome), and neutral Keima as a green fluorescent signal at higher pH (mitochondria)^59^. Fluorescence of mt-Keima was imaged in two channels via two sequential excitations (458 nm, green; 561 nm, red) and using a 570- to 695-nm emission range. The level of mitophagy was defined as the total number of red pixels divided by the total number of all pixels.

### In vivo protein synthesis measurements

In vivo protein synthesis was measured by using the SUnSET technique^25,60^. Mice were anesthetized and then given an intraperitoneal injection of 0.040 μmol/g puromycin dissolved in 100 μl of PBS. At exactly 30 min after injection, muscles were collected and frozen in liquid N_2_ for western blot analysis. A mouse IgG2a monoclonal anti-puromycin antibody (clone 12D10, 1:5000) was used to detect puromycin incorporation.

### Contractile force measurements

In vivo gastrocnemius force measurements were performed as described previously^61^. Briefly, mice were anesthetized, and stainless-steel electrodes wires were placed on either side of the sciatic nerve. Torque production of the plantar flexors was measured using a muscle lever system (Model 305c; Aurora Scientific, Aurora, ON, Canada). The force–frequency curves were determined by increasing the stimulation frequency in a stepwise manner, pausing for 30 s between stimuli to avoid effects due to fatigue. After force measurements, animals were killed by cervical dislocation and muscles were dissected and weighted. Force was normalized to the muscle mass as an estimate of specific force.

Ex vivo, EDL and SOL muscles were dissected in warm oxygenated Krebs solution and mounted between a force transducer (SI-H Force Transducer World Precision Instruments, Inc., Sarasota, FL, USA) and a micromanipulator-controlled shaft in a small chamber where oxygenated Krebs solution was continuously circulated. The temperature was kept constant at 25 °C.

After optimizing the stimulation conditions and muscle length, the responses to a single stimulus (twitch) or to a series of stimuli at increasing rates producing unfused or fused tetani were recorded. Time-to-peak tension, time-to-half relaxation, and peak-tension were measured in single twitches. Tension was measured in unfused and fused maximal tetani. Fatigue was induced by 120 s of repetitive stimulation applying every 2 s a train of 0.5 s duration (duty ratio 1/4).

### In vivo FDB electroporation

Electroporation experiments were performed on FDB muscles from wild-type and knockout animals. The animals were anesthetized by an intraperitoneal injection of xylazine (Xilor) (20 mg/Kg) and Zoletil (10 mg/Kg). 7 μl of hyaluronidase (2 mg/ml) (Sigma-Aldrich) were injected in the feet of anesthetized mice to soften muscle tissue underneath the epidermis. After 50 min, we injected 15 μg of plasmid DNA (4mitoD3cpv Cameleon; mt-Keima probe; D1-ER Cameleon; shRNA against MCU; DRP1-YFP) and after 10 min electric pulses were applied by two stainless needles placed at 1 cm from each other (100 V/cm) (100 Volts/cm, 20 pulses, 1 s intervals). Muscles were analyzed 10 days later. No evidence of necrosis or inflammation were observed after the transfection procedure.

### Autophagic flux quantification

We monitored autophagic flux in basal condition using colchicine^62^. Briefly, inducible transgenic mice were treated with 0.4 mg/kg of colchicine or the vehicle by intraperitoneal injection. The treatment was repeated twice every 12 h prior to muscle harvesting.

### Plasma FGF21 and IGF1 measurements

Plasma was obtained from blood collected from controls and DRP1-null mice. Blood FGF21 levels were determined using rat/mouse FGF21 enzime-linked immunosorbent ELISA-Kit (Merck Millipore, EZRMFGF21-26K). Blood IGF1 levels were measured using mouse/rat IGF1 immunoassay (MG100, R&D Systems) and following the manufacturer’s instructions. The data are expressed in pg/ml.

### Statistical analysis

All data are expressed as means ± SEM of independent experiments. Statistical analysis was performed using two-tailed Student’s *t* test or two-way analysis of variant (ANOVA). When ANOVA revealed significant differences, further analysis was performed using Bonferroni’s multiple comparison test. Differences between groups were considered statistically significant for *p* < 0.05.

### Reporting summary

Further information on research design is available in the Nature Research Reporting Summary linked to this article.

## Supplementary information

Supplementary Information

Reporting Summary

## Data Availability

The authors declare that all data supporting the findings of this study are available within the paper and/ or its supplementary information files.
