# Supplementary material for: DRP1-mediated mitochondrial shape controls calcium homeostasis and muscle mass
Source: Nat Commun. 2019 Jun 12;10:2576. doi: 10.1038/s41467-019-10226-9 (PMC6561930; doi:10.1038/s41467-019-10226-9)
Supplement: Supplementary file 1 — Supplementary Information [file 41467_2019_10226_MOESM1_ESM.pdf]

DRP1-MEDIATED MITOCHONDRIAL SHAPE CONTROLS CALCIUM HOMEOSTASIS AND MUSCLE MASS

Favaro et al.

Supplementary Figure 1

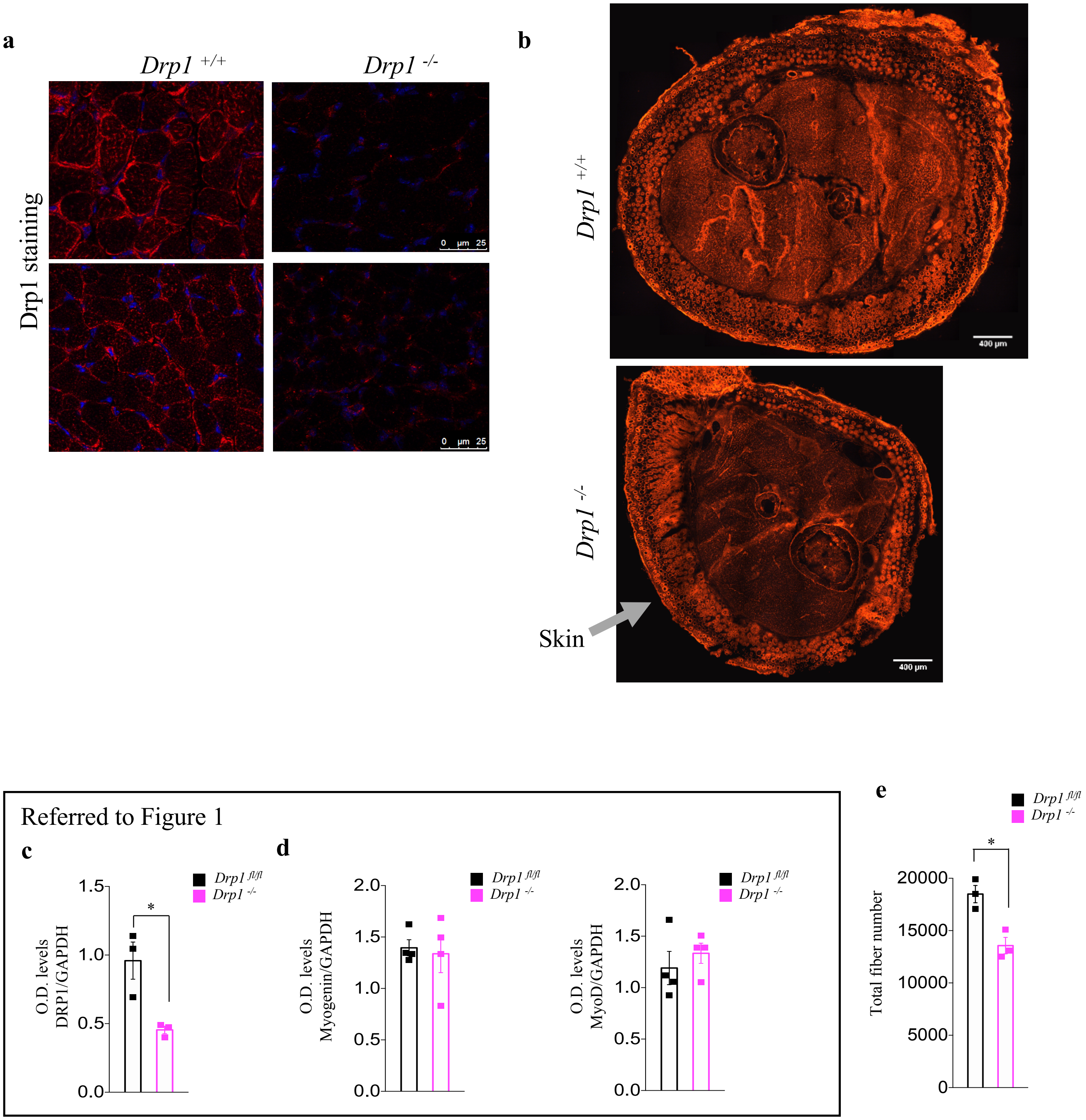

Supplementary Figure 1.

a) DRP1 staining confirming the deletion of DRP1 protein in muscles from MLC-DRP1 KO compared to control. b) DRP1 immunofluorescence in hindlimb cross-section. The expression of DRP1 in KO is restricted to skin, vessels and bones, while the signal is absent in muscle. c-d) Densitometric quantification of the western blots related to Figure 1. Data represent average ± SEM. *p≤0.05. e) Quantification of fibers number present in hind limb muscles

Supplementary Figure 2

a

	A	B
	**Mitochondria Volume/Total volume (%)**	**Average size of apparently normal mitochondria ($\mu m^2 x10^{-3}$)**
**Drp1 $^{fl/fl}$**	8.0 ± 0.3	157 ± 10
**Drp1 $^{-/-}$**	7.6 ± 0.3	270 ± 40*

c

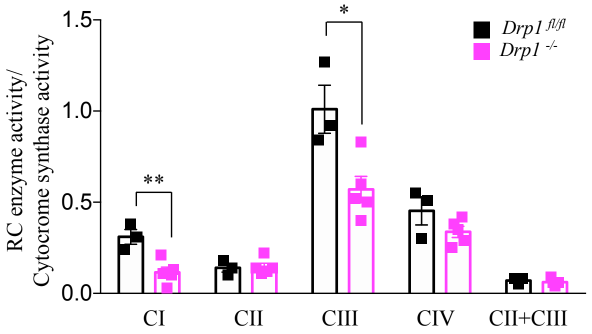

b

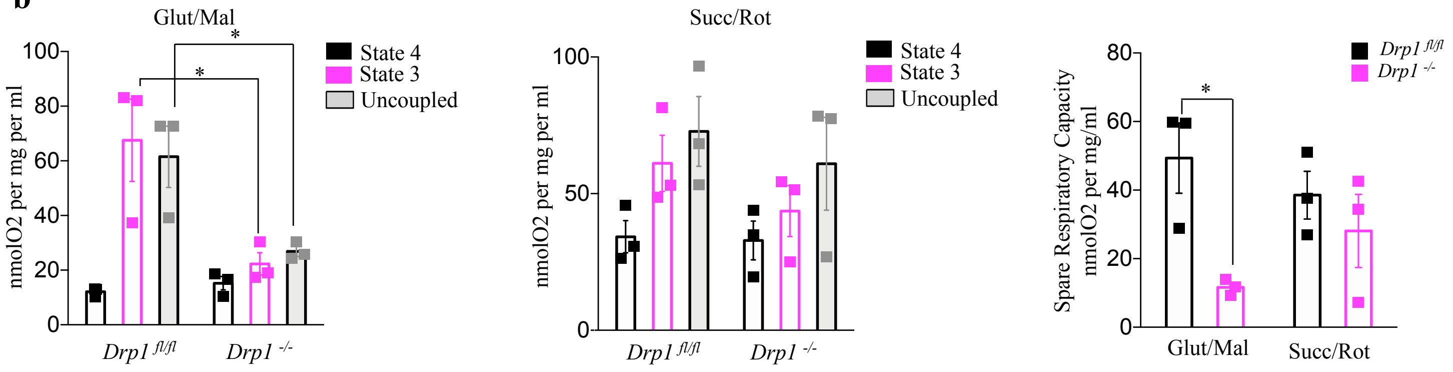

d

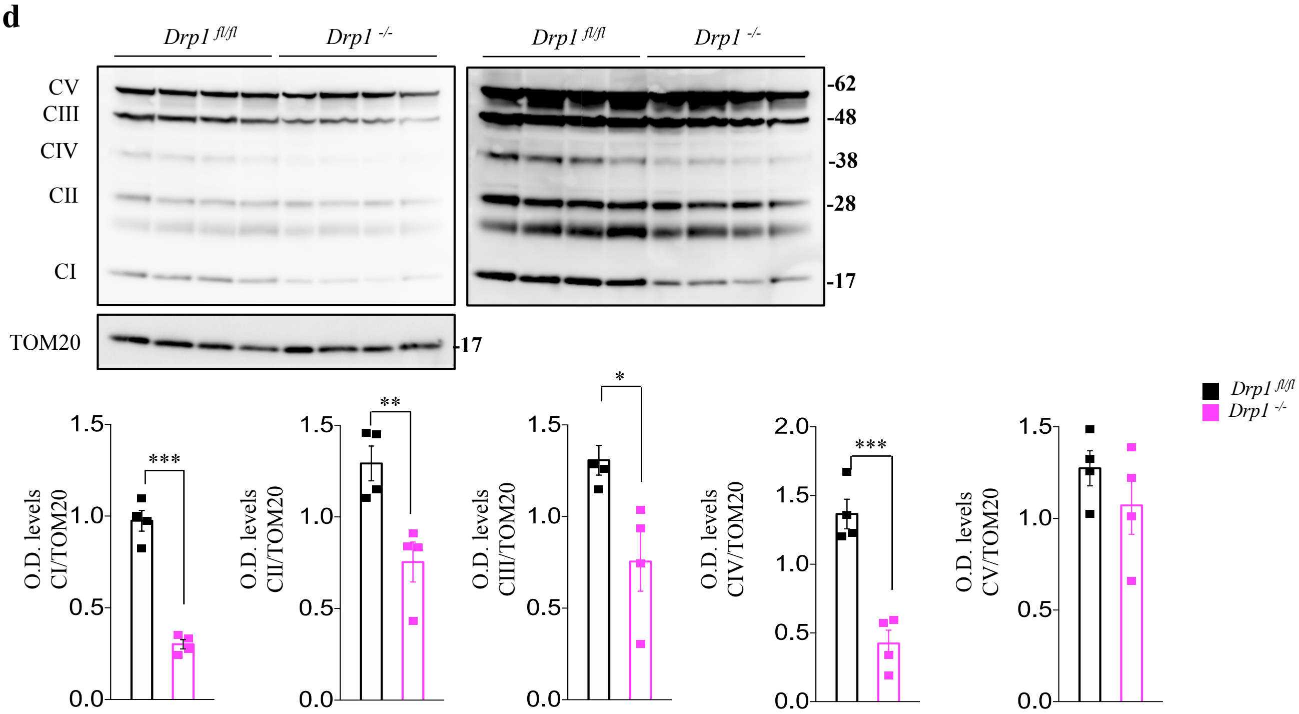

Referred to Figure 2

e

f

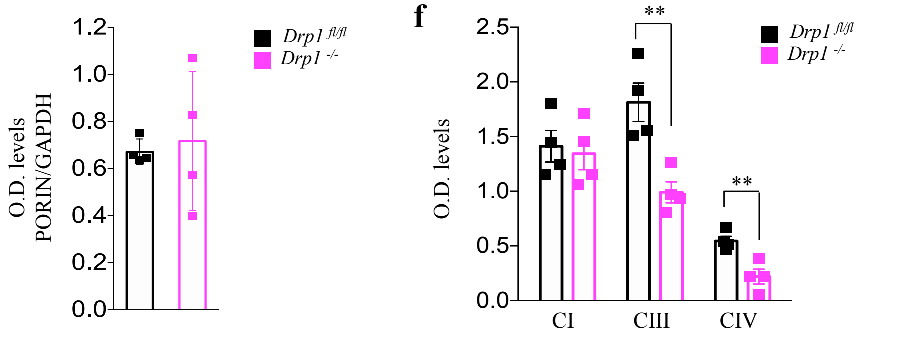

Supplementary Figure 2.

a) Quantification of the relative fiber volume occupied by mitochondria in the MLC1f-DRP1 mouse model. The cytosolic area occupied by mitochondria is slightly (but not significantly) decreased in *Drp1*$^{-/-}$ fibers (column A). However, morphometric analysis shows a significant change in the average size of mitochondria: in *Drp1*$^{-/-}$ fibers mitochondria are significantly larger in size (column B). Data are shown as mean ± SEM (*p < 0.01). Sample size: column A, *Drp1*$^{fl/fl}$ 10/15 fibers, 1 micrograph/fiber; DRP1$^{-/-}$ 10/15 fibers, 1 micrograph/fiber. Column B, *Drp1*$^{fl/fl}$ 5 fibers, 1 micrograph/fiber; n=132 mitochondria analyzed; DRP1$^{-/-}$: 5 fibers, 1 micrograph/fiber; n=219 mitochondria analyzed. b) Complex I (glutamate/malate) and Complex II (succinate/rotenone) dependent oxygen consumption in isolated mitochondria from *Drp1*$^{fl/fl}$ and *Drp1*$^{-/-}$ muscles. Glutamate supported State 3 (ADP), uncoupled respiration and the spare respiratory capacity are decreased in *Drp1*$^{-/-}$ mitochondria. Data represent average ± SEM (n=3 each condition). c) CI and CIII enzyme activity in mitochondria isolated from MLC-DRP1 KO muscles is decreased compared to control (WT n=3; KO n=5). d) Immunoblot and densitometric analysis of mitochondrial respiratory complexes. CI, CII, CIII and CIV are downregulated in *Drp1*$^{-/-}$ muscles (n=4 each condition). e-f) Densitometric quantification of the western blots related to Figure 2. Data represent average ± SEM. *p≤0.05; **p≤0.01; ***p≤0.001.

Supplementary Figure 3

a

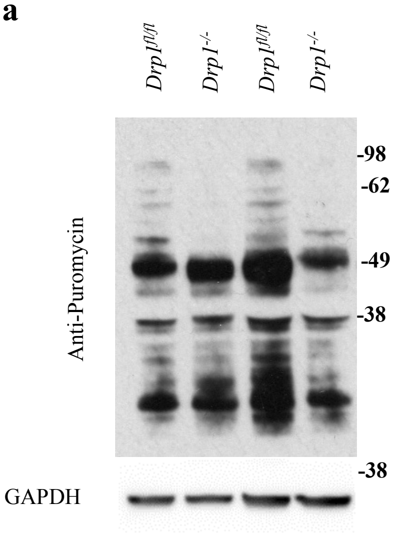

b

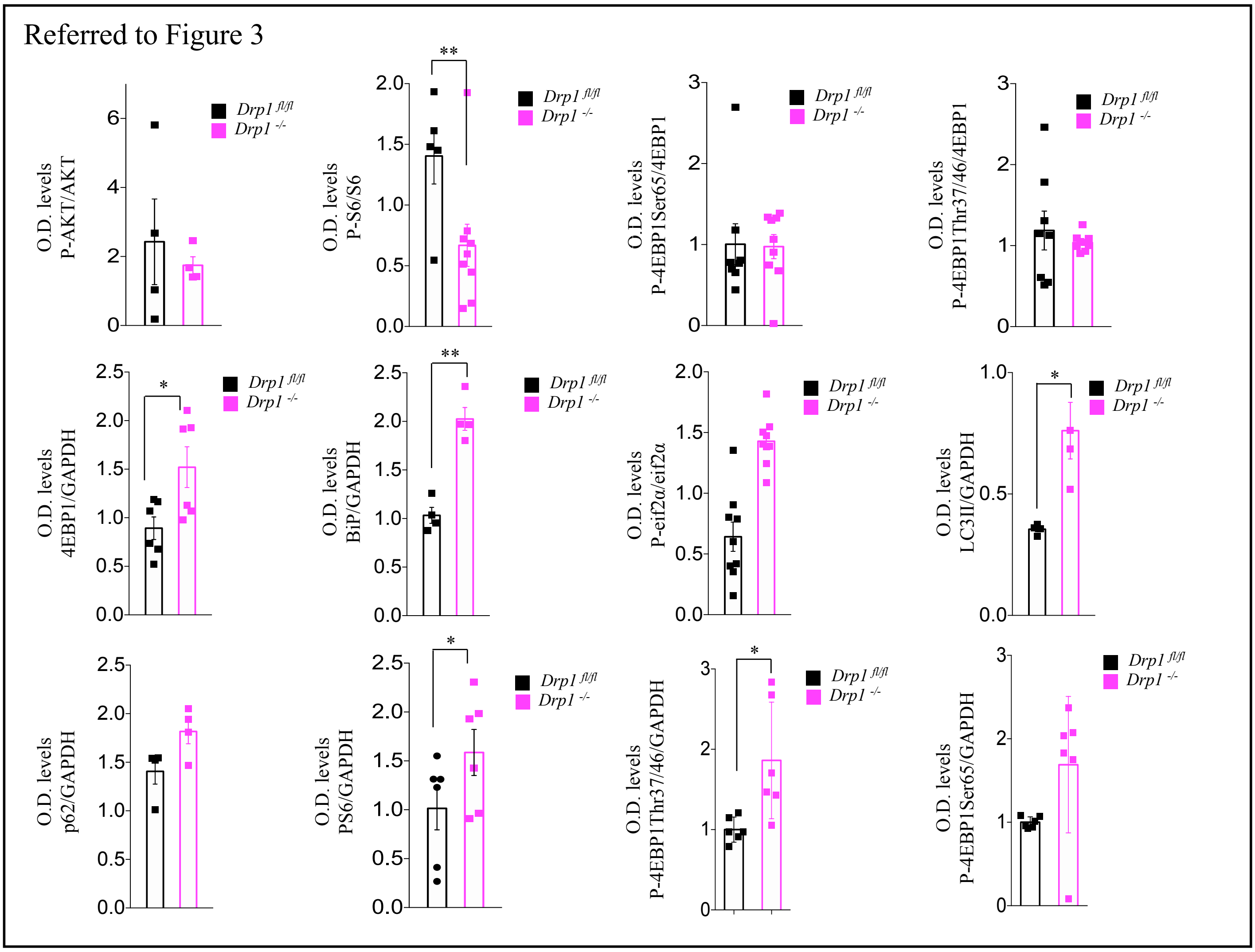

Supplementary Figure 3.

a) *In vivo* SUnSET technique shows a significant reduction of protein synthesis in DRP1-ablated muscles. b) Densitometric quantification of the western blots related to Figure 3. Data represent average ± SEM. *p≤0.05; **p≤0.01.

Supplementary Figure 4

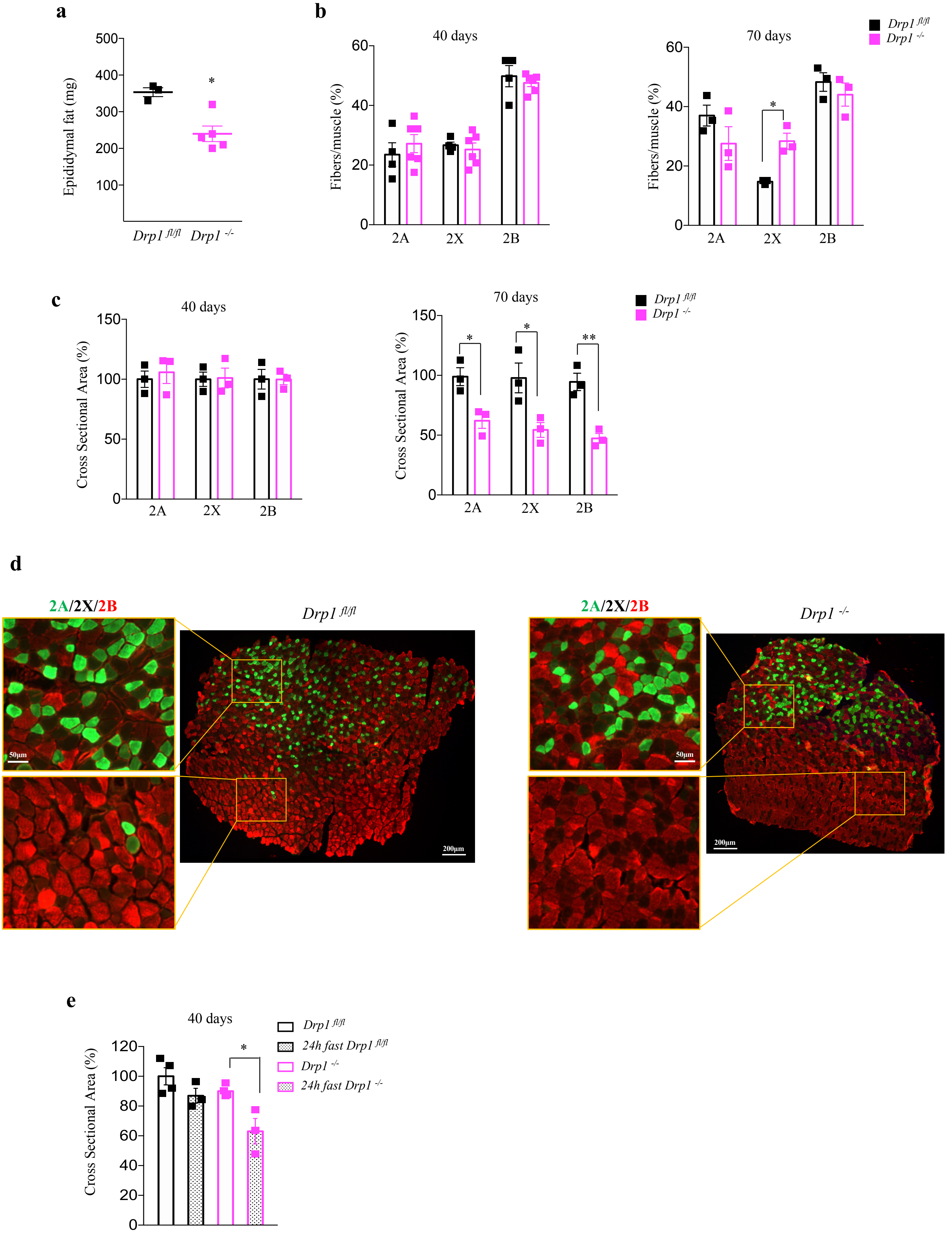

Supplementary Figure 4.

a) Epididymal fat in HSA-DRP1 KO mice is decreased compared to control (WT n=4; KO n=5). b) Fiber type distribution in Tibialis Anterior muscles. After 70 days of DRP1 deletion number of 2X fibers increased (n=3 for each condition), while there are no differences after 40 days of tamoxifen treatment (WT n=4; KO n=6). c) Cross-Sectional Area analysis in different fiber type in HSA-DRP1 model. After 70 days of treatment 2A, 2X and 2B fibers are significantly smaller compared to control (n=3 for each condition). d) Representative immunostaining showing different fiber type distribution in Tibialis Anterior from control and KO mice (70 days). e) After 24 hours of fasting, myofiber cross-sectional area in Tibialis Anterior muscle from KO mice (40 days of treatment) decreased compared to fed KO mice (n=3 each condition). Data represent average ± SEM. *$p \leq 0.05$; **$p \leq 0.01$.

Supplementary Figure 5

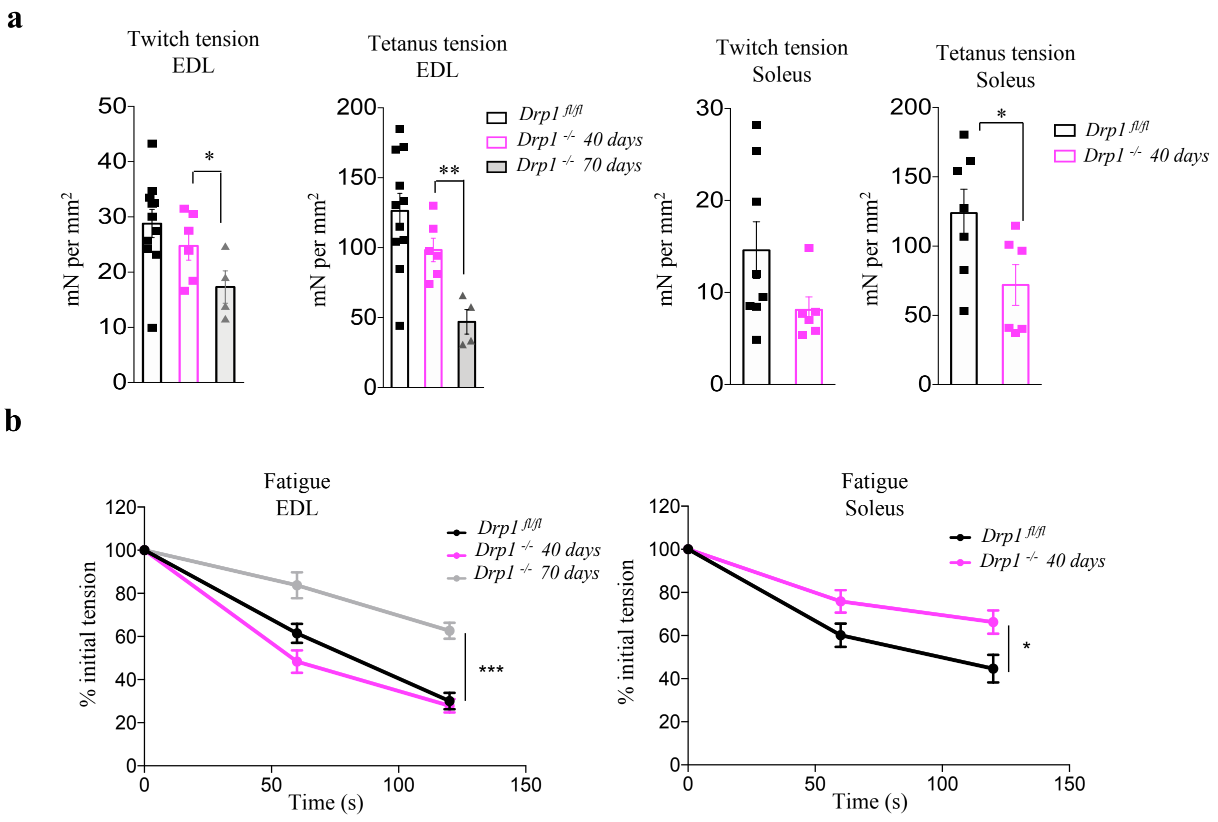

Supplementary Figure 5.

a) Tension developed by EDL and Soleus muscles *ex vivo* in response to single stimulus (twitch) or a train of high frequency stimulation sufficient to induce fused tetanus (for soleus 80-90 Hz, for EDL 120-150 Hz). Tension development is significantly reduced in both, twitch and tetanus in Drp1-null muscles.

b) fatigue induced by repetitive stimulation with brief maximal tetani (0.5 s duration every 2 s) in EDL and Soleus. Fatigue is expressed by the reduction of tension expressed in % of the tension developed during the first tetanus. Fatigue is significantly reduced in EDL after 70d and in Soleus after 40 d of tamoxifen treatment. Data represent average ± SEM. *$p \leq 0.05$; **$p \leq 0.01$; ***$p \leq 0.001$.

Supplementary Figure 6

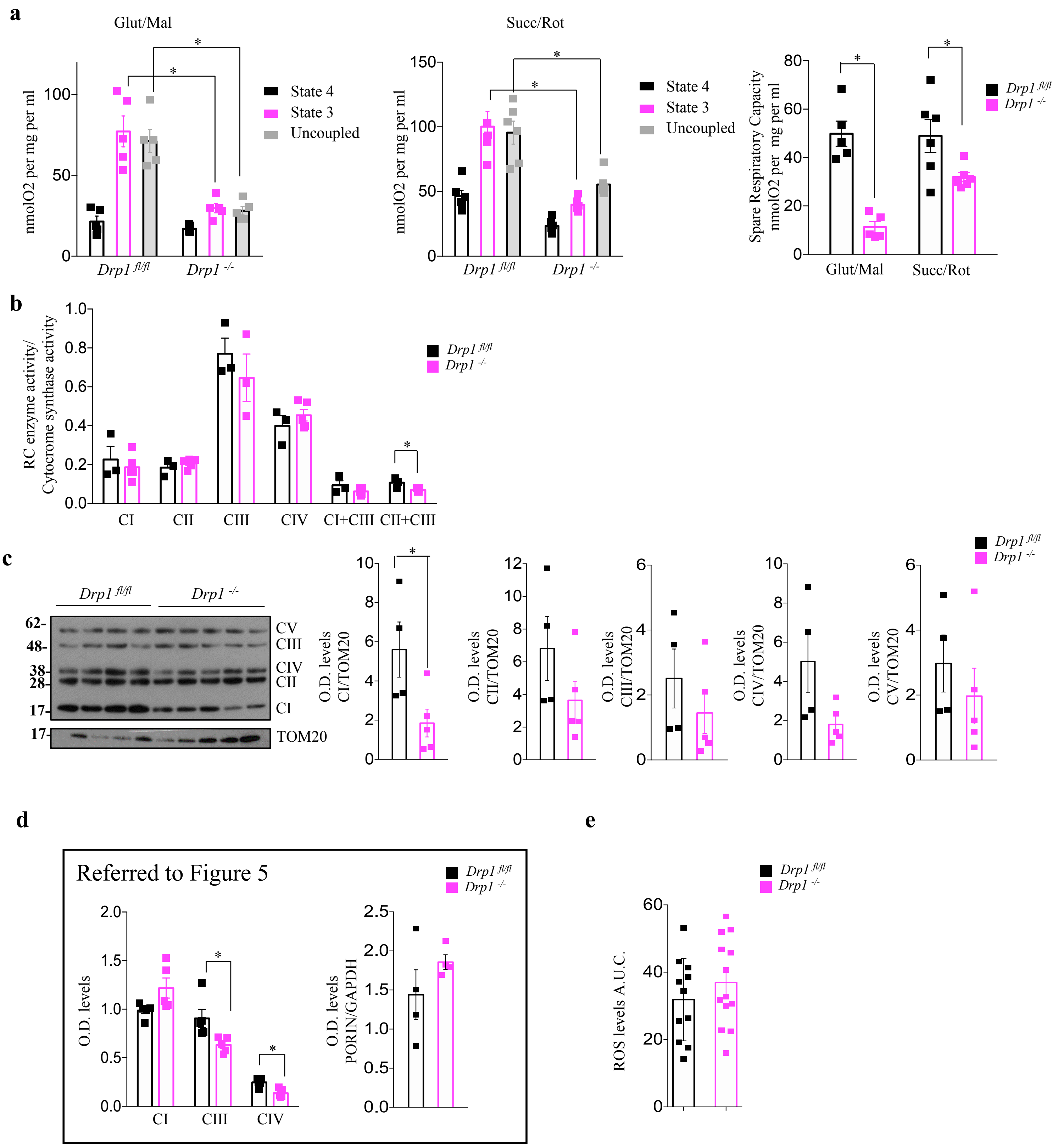

Supplementary Figure 6.

a) Complex I (glutamate/malate) and Complex II (succinate/rotenone) dependent oxygen consumption in isolated mitochondria from *Drp1*$^{fl/fl}$ and *Drp1*$^{-/-}$ muscles. Glutamate supported State 3 (ADP), Succinate supported State 3, uncoupled respiration and the spare respiratory capacity are decreased in *Drp1*$^{-/-}$ mitochondria. Data represent average ± SEM (Glut/Mal n=5 each condition; Succ/Rot n=6 each condition). b) Respiratory complex single enzyme activity did not change after Drp1 deletion, while CII+CIII complexes activity is reduced in mitochondria from HSA-DRP1 KO mice. c) Immunoblot representing mitochondria complexes levels. CI is significantly decreased in muscles from adult KO animals (WT n=4; KO n=5). d) Densitometric quantification of the western blots related to Figure 5. Data represent average ± SEM. *p≤0.05. e) Quantification of mitochondrial ROS production in FDB isolated fibers shows no difference between WT and KO mice. Data represent average ± SEM. *p≤0.05.

Supplementary Figure 7

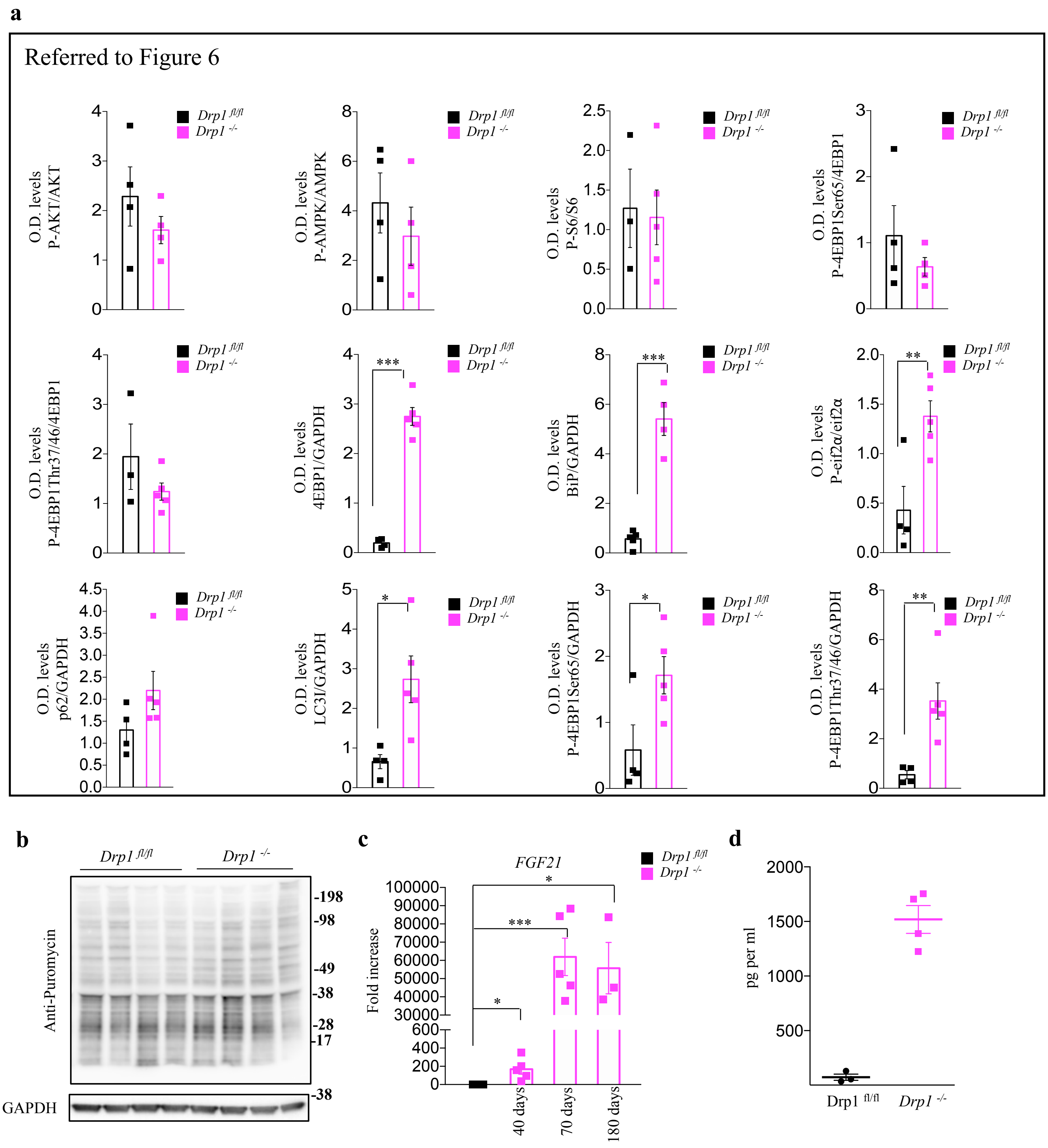

Supplementary Figure 7.

a) Densitometric quantification of the western blots related to Figure 6. b) Total protein extracts from adult muscles treated with puromycin were immunoblotted with reported antibodies (n=4 mice each condition). c) RT-PCR analysis show increased FGF21 expression levels in *Drp1*$^{-/-}$ in three different timepoints. Data represent average ± SEM. *p≤0.05; **p≤0.01; ***p≤0.001. d) Quantification of FGF-21 in the blood of adult mice after 70 days of tamoxifen treatment (WT, n=3; KO, n=4).

Supplementary Figure 8

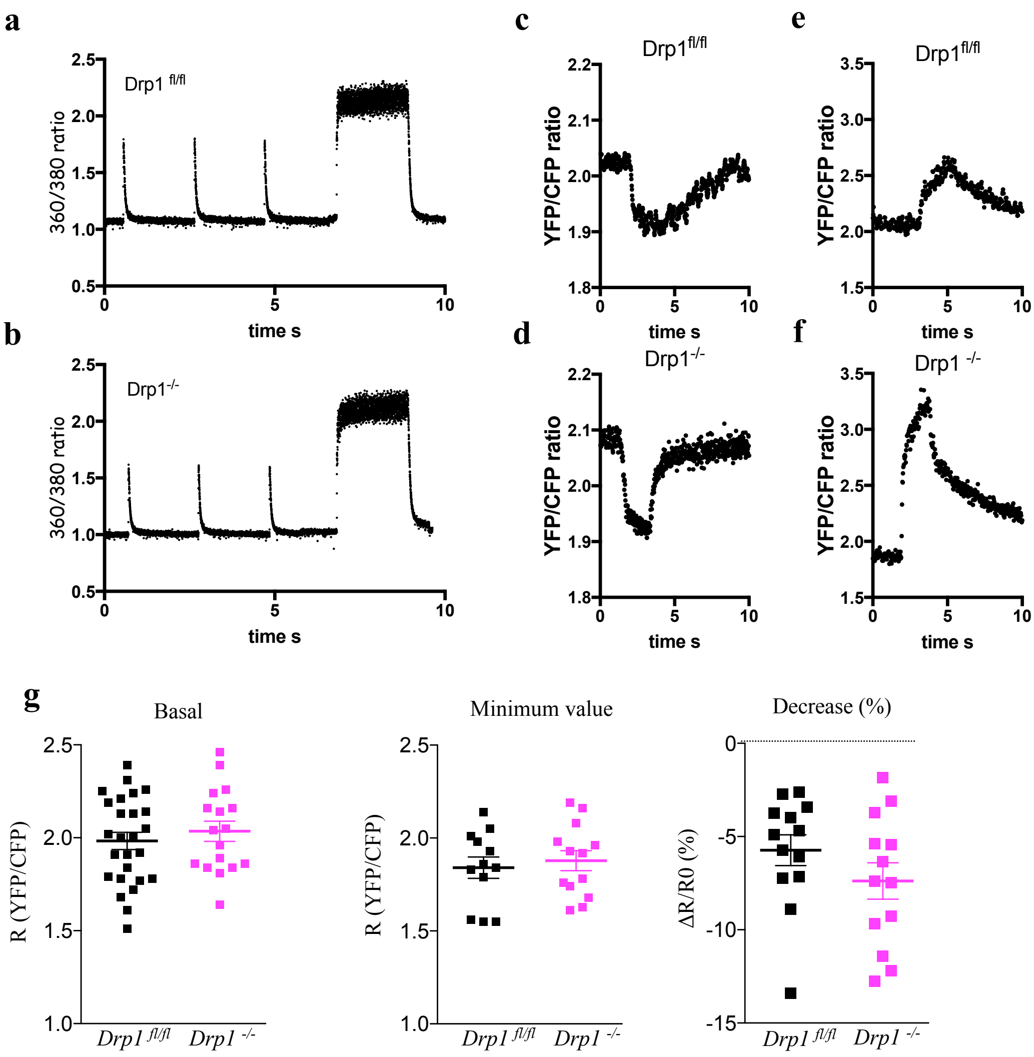

Supplementary Figure 8.

a) and b) Representative traces of cytosolic calcium (Fura 2 ratio) during electrical stimulation at low frequency (0.5 Hz) and high frequency (tetanus at 60 Hz, 2 s duration) in Drp1$^{fl/fl}$ (a) and Drp1$^{-/-}$ (b) single fibers.

c) and d) Representative transients of free calcium concentration in sarcoplasmic reticulum as measured by ERD1 cameleon fluorescence ratio during a train of high frequency stimulation (2 s at 60 Hz) in Drp1$^{fl/fl}$ and Drp1$^{-/-}$

e) and f) Representative transients of free calcium concentration in mitochondrial matrix as measured by mtD3cpv cameleon fluorescence ratio during a train of high frequency stimulation (2 s at 60 Hz) in Drp1$^{fl/fl}$ and Drp1$^{-/-}$

g) SR free Ca^{2+} levels (determined with cameleon ER-D1 in basal condition (WT n=26; KO n=17) and after high frequency (tetanus at 60 Hz, 2 s duration) electrical stimulation are not significantly affected after DRP1 deletion.

Supplementary Figure 9

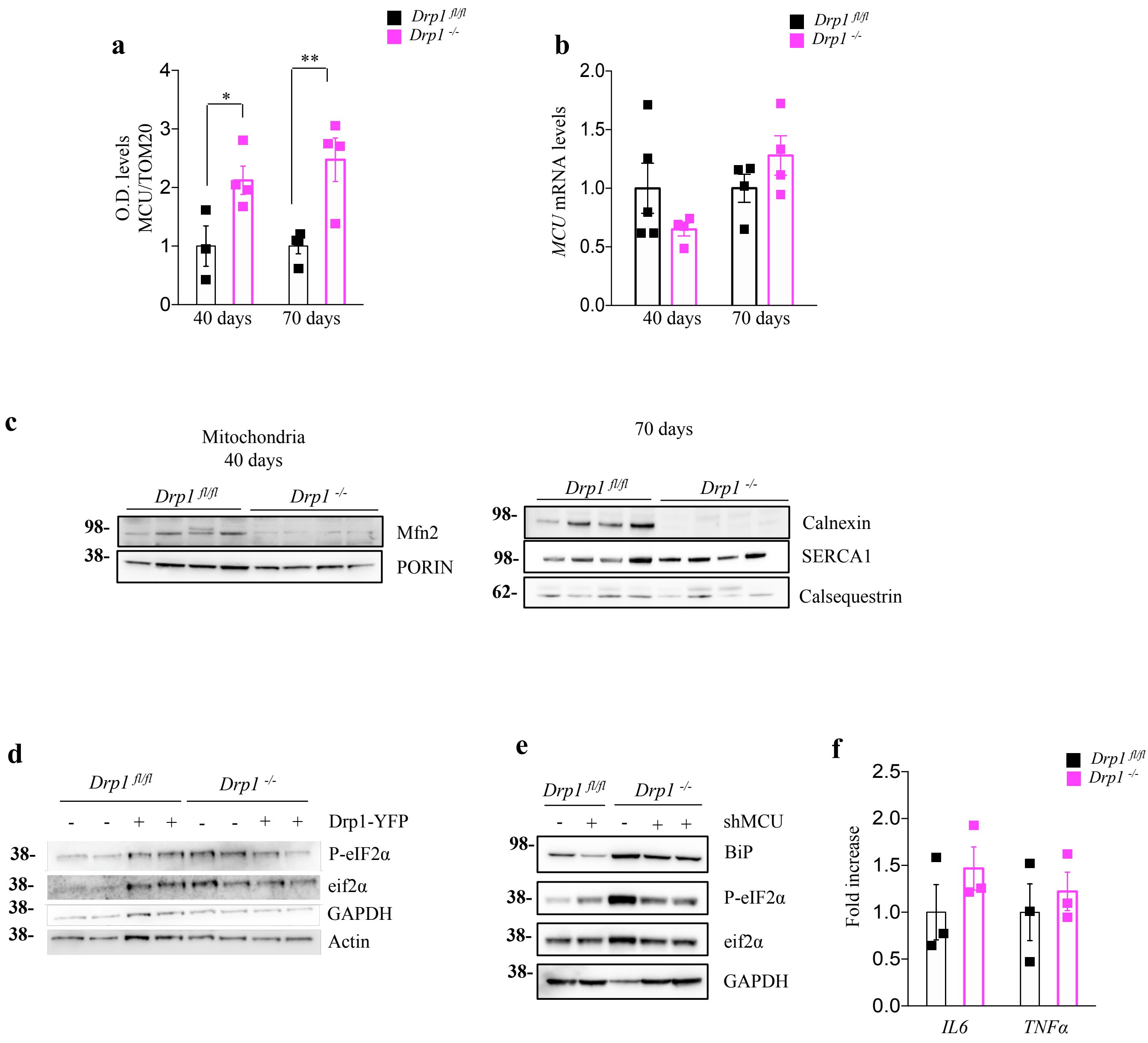

Supplementary Figure 9.

a) O.D. levels related to Figure 7e. Data represent average ± SEM. *p≤0.05; **p≤0.01. b) RT-PCR analysis of transcriptional levels of MCU show no differences in KO muscles (n=4 each condition). Data represent average ± SEM. c) Western Blot analysis in isolated mitochondria (40 days) show a decrease in Mfn2 protein levels in *Drp1*$^{-/-}$ mice. Immunoblots from muscles (70 days) showing a decrease in calnexin levels after DRP1 deletion. d) DRP1 rescue experiment (Drp1-YFP) leads to a reduction of ER stress markers in KO muscles. e) 2 months of MCU silencing in muscle (shMCU) leads to decrease of major ER stress markers protein levels in KO muscles. f) RT-PCR analysis show no differences in TNFα and IL-6 levels between WT and DRP1 KO muscles (n=3).

Supplementary Figure 10

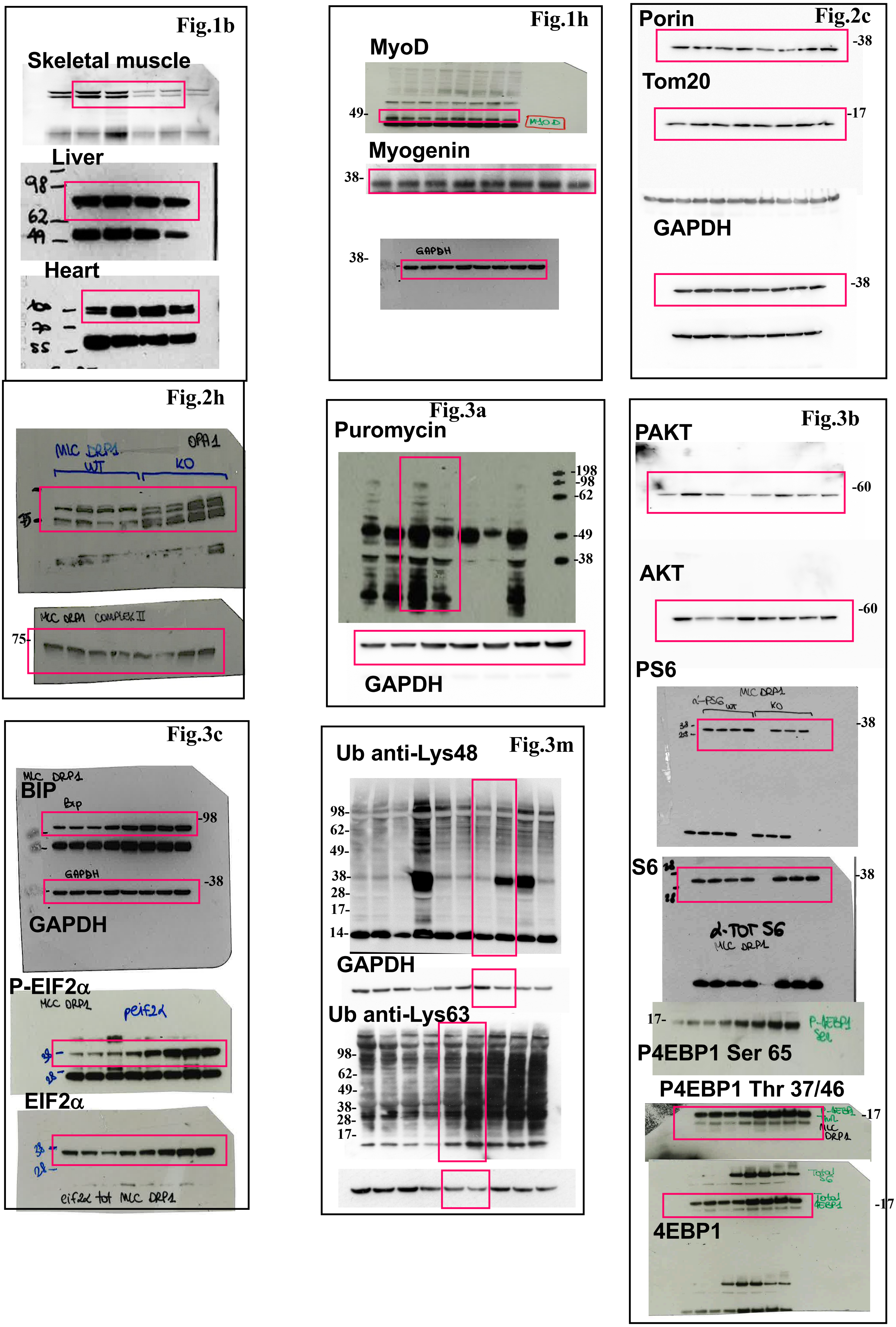

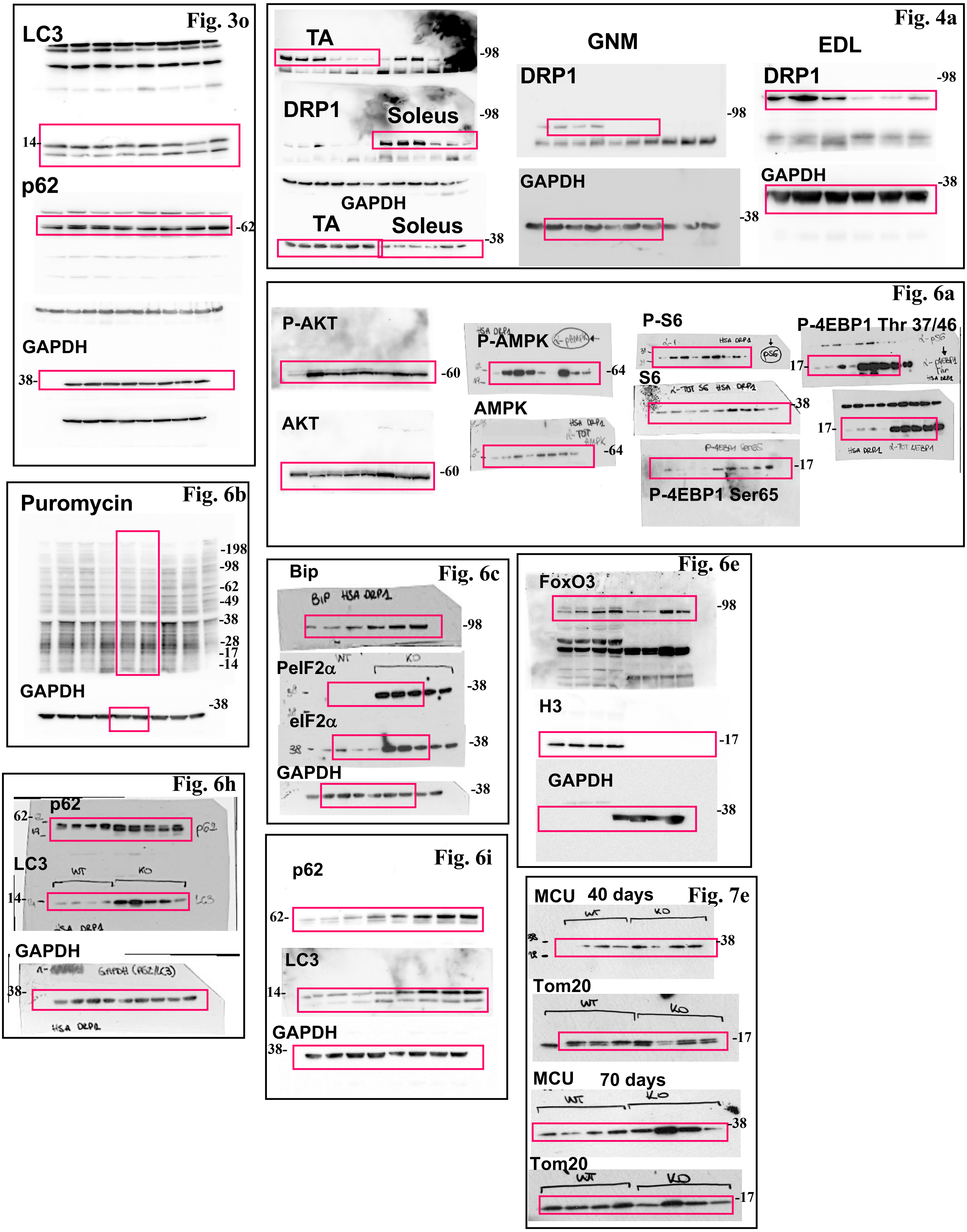

Supplementary Figure 10. Uncropped blots of main figures

Supplementary Tables

Supplementary Table 1

	A	B	C	D
	Mitochondria volume / total volume %	No. of mitochondria /100 μm^2	No. of damaged mitochondria /100 μm^2	Average size of apparently normal mitochondria μm^{2}x10^{-3}
Drp1 $^{fl/fl}$	4.1 ± 0.3	44.4 ± 1.2	1.0 ± 0.2 (2%)	70.6 ± 2.0
Drp1 $^{-/-}$	7.2± 0.1*	37.4 ± 1.0*	3.8 ± 0.4 (10%)	111.6 ± 5.6*

Supplementary Table 1. Quantitative analyses of mitochondria. Mitochondria in EDL muscle fibers from *Drp1$^{-/-}$* mice occupy a larger volume of the fiber (column A), even if their number is slightly reduced (column B). Mitochondria exhibiting structural abnormalities are more frequent in *Drp1$^{-/-}$* than in *Drp1$^{fl/fl}$* fibers (column C). Parenthesis in column C: n. of damaged mitochondria expressed as percentage of the total. Finally, even those mitochondria that are apparently normal are significantly larger in size, an indication of mitochondria being swollen (column D). Columns A: 15 fibers, 10 micrograph/fiber; *Drp1$^{-/-}$* : 20 fibers, 10 micrograph/fiber. Column B: *Drp1$^{fl/fl}$*: 10 fibers, 2 micrograph/fiber; *Drp1$^{-/-}$* : 10 fibers, 2 micrograph/fiber. Column C: *Drp1$^{fl/fl}$* n. 2338 mitochondria analyzed; *Drp1$^{-/-}$* n. 2727 mitochondria analyzed. Column D: *Drp1$^{fl/fl}$*, n. 563 mitochondria analyzed; *Drp1$^{-/-}$* n. 706 mitochondria analyzed. Data are shown as mean ± SEM (*p < 0.01).

Supplementary Tables

Supplementary Table 2

	A	B	C	D
	No. of CRUs /100 μm^2	Oblique/ Longitudinal CRUs (%)	Dyads (%)	No. of Mito-CRU pairs /100 μm^2
Drp1 $^{fl/fl}$	69.1 ± 1.4	1.1 ± 0.4	3.7 ± 2.1	33.7 ± 1.5
Drp1 $^{-/-}$	57.0 ± 1.8*	2.4 ± 0.7*	12.6 ± 1.3*	24.0 ± 1.0*

Supplementary Table 2. Quantitative analyses of Calcium Release Units (CRUs). In EM micrographs we determined: i) number per area of CRUs (column A); ii) orientation (oblique/longitudinal triads; column B) and iii) percentage of incomplete triads expressed as percentages over total number of CRUs (dyads; column C); and iv) mitochondria-CRUs pairs (column D). In Drp1$^{-/-}$ fibers the number/area of CRUs is significantly decreased compared to controls (column A), with a concomitant increase of miss-oriented and incomplete triads (columns B and C). The decrease in CRUs (together with the decrease in mitochondria (see Table 1) results is a significant reduction of mitochondria/CRU pairs (column D).

Samples size: *Drp1$^{fl/fl}$*: 3 mice, 15 fiber, 10 micrograph/fiber; *Drp1$^{-/-}$* : 4 mice, 20 fibers, 10 micrograph/fiber. Data are shown as mean ± SEM (*p < 0.01).

Supplementary Tables

Supplementary Table 3

**Phenotype/Signaling**	**MLC DRP1 (conditional)**	**HSA DRP1 (inducible)**
Muscle Atrophy	Yes	Yes
Myofiber loss/Degeneration	Yes	Yes
Force drop/Weakness	Not Done	Yes
Mitochondria Dysfuction	Yes	Yes
Increased Mitochondrial Volume	Yes	Yes
ER stress (UPR)	Yes	Yes
Decreased Protein Synthesis	Yes	No
Increased UPS	Yes	Yes
Mild or No increased autophagy	Yes	Yes
Inhibition of Mitophagy	Not Done	Yes
Oxidative stress	Not Done	No
Calcium Dysregulation	Not Done	Yes

Supplementary Table 3. Comparison of the major features present in MLC-DRP1 (conditional) and HSA-DRP1 (inducible muscle-specific) mouse models.

Supplementary Tables

Supplementary Table 4

	Forward primer (5'-3')	Reverse primer (3'-5')
**Drp1**	TCAGATCGTCGTAGTGGGAA	TCTTCTGGTGAAACGTGGAC
**ATF4**	TCCTGAACAGCGAAGTGTTG	ACCCATGAGGTTTCAAGTGC
**GADD34**	AGAGAAGACCAAGGGACGTG	CAGCAAGGAATGGACTGTG
**CHOP**	GCTGGAAGCCTGGTATGAG	ATGTGCGTGTGACCTCTGTT
**FGF21**	ATGGAATGGATGAGATCTAGAGTTGG	TCTTGGTGGTCATCTGTGTAGAGG
**Atrogin1**	GCAAACACTGCCACATTCTCTC	CTTGAGGGGAAAGTGAGACG
**MuRF1**	ACCTGCTGGTGGAAAACATC	ACCTGCTGGTGGAAAACATC
**MUSA1**	TCGTGGAATGGTAATCTTGC	CCTCCCGTTTCTCTATCACG
**Smart1**	TCAATAACCTCAAGGCGTTC	GTTTTGCACACAAGCTCCA
**Fbxo31**	GTATGGCGTTTGTGAGAACC	AGCCCCAAAATGTGTCTGTA
**Trim37**	ACACTGAGAACGAGGACAG	CAACAAATTTCAGGGACCAG
**Itch**	CCACCCACCCCACGAAGACC	CTAGGGCCCGAGCCTCCAGA
**Beclin1**	TGGAAGGGTCTAAGACGT	GGCTGTGGTAAGTAATGGA
**LC3**	CACTGCTCTGTCTTGTGTAGGTTG	TCGTTGTGCCTTTATTAGTGCATC
**Bnip3**	TTCCACTAGCACCTTCTGATGA	GAACACCGCATTTACAGAACAA
**p62**	CCCAGTGTCTTGGCATTCTT	AGGGAAAGCAGAGGAAGCTC
**GabarapL**	CATCGTGGAGAAGGCTCCTA	ATACAGCTGGCCCATGGTAG
**CathepsinL**	GTGGACTGTTCTCACGCTCAAG	TCCGTCCTTCGCTTCATAGG
**PGC1α**	GGAATGCACCGTAAATCTGC	TTCTCAAGAGCAGCGAAAGC
**Mfn1**	GCTGTCAGAGCCCATCTTTC	CAGCCCACTGTTTTCCAAAT
**Mfn2**	ATGTTACCACGGAGCTGGAC	AACTGCTTCTCCGTCTGCAT
**Opa1**	ATACTGGGATCTGCTGTTGG	AAGTCAGGCACAATCCACTT
**Fis1**	AAGTATGTGCGAGGGCTGT	TGCCTACCAGTCCATCTTTC
**IL6**	TAGTCCTTCCTACCCCAATT	TTGGTCCTAAGCCACTCCTT
**TNFα**	CACAAGATGCTGGGACAGT	TCCTTGATGGTGGTGCATGA
**GAPDH**	CACCATCTTCCAGGAGCGAG	CCTTCTCCATGGTGGTGAAGAC

Supplementary Table 4. List of primers used for Real-Time PCR analyses.

Supplementary Tables

Supplementary Table 5

**Antibody**	**Customer**	**Dilution**
Rabbit anti-phospho-Akt (Ser473)	Cell Signaling #3787	1:1000
Rabbit anti-Akt	Cell Signaling #9272	1:1000
Rabbit anti-phospho-AMPK (Thr172)	Cell Signaling #2535	1:1000
Rabbit anti-AMPK	Cell Signaling #2532	1:1000
Rabbit anti-phospho-S6	Cell Signaling #2215	1:1000
Rabbit anti-S6	Cell Signaling #2217	1:1000
Rabbit anti-phospho-4EBP1 (Thr37/46)	Cell Signaling #9459	1:2000
Rabbit anti-phospho-4EBP1 (Ser65)	Cell Signaling #9455	1:1000
Rabbit anti-4EBP1	Cell Signaling #9452	1:2000
Mouse anti-BiP/GRP78	BD 610979	1:5000
Rabbit anti-phospho-eif2α	Abcam ab 32157	1:1000
Rabbit anti-eif2α	Cell Signaling #9722	1:1000
Rabbit anti-p62	Sigma P0067	1:2000
Rabbit anti-LC3	Sigma L7543	1:1000
Mouse anti-Drp1	BD 611738	1:2000
Mouse anti-GAPDH	Abcam ab8245	1:10000
Mouse anti-puromycin	Hybridoma Bank PMY-2A4	1:5000
Rabbit anti-MyoD	Santa Cruz sc-32758	1:1000
Rabbit anti-Myogenin	Millipore MAB3876	1:1000
Mouse anti-Porin	Santa cruz Sc-11415	1:10000
Rabbit anti-MCU	Sigma HPA016480	1:2000
Mouse anti-OPA1	BD 612606	1:2000
Mouse anti-ubiquitinylated proteins clone FK2	Millipore 04-263	1:5000
Rabbit anti-Ubiquitin Lys63-Specific clone Apu3	Millipore 05-1308	1:1000
Rabbit anti-TOM20	Abcam ab14734	1:10000
Mouse anti-NDUFB8	Mol Probes 459210	1:5000
Mouse anti-CORE2	Mitoscience MS304	1:5000
Mouse anti-COXI	Mitosciences MS404	1:5000
Rabbit anti-FoxO3	Cell signaling #9946	1:1000
Rabbit anti-phospho-histone H3 (Ser10)	Cell signaling #3377	1:10000
Goat anti mouse Cy3	Jackson Laboratories 115-165-003	1:200
Goat anti mouse IgG	Biorad 1706516	1:2000
Goat anti rabbit IgG	Biorad 1706515	1:2000

Supplementary Table 5

**Antibody**	**Customer**	**Dilution**
Mouse anti-Mfn2	Abcam 56889	1:2000
Total OXPHOS Rodent WB Antibody Cocktail	Abcam 110413	1:5000
Rabbit anti-Calnexin	Abcam 10286	1:1000
Mouse anti-SERCA ATPase (VE12IG9)	ThermoFischer Scientific MA3-912	1:5000
Mouse anti-Calsequestrin (VIIID12)	ThermoFischer Scientific MA3-912	1:1000

Supplementary Table 5. List of primary and secondary antibodies.
